# Fungi hijack a ubiquitous plant apoplastic endoglucanase to release a ROS scavenging β-glucan decasaccharide to subvert immune responses

**DOI:** 10.1093/plcell/koac114

**Published:** 2022-04-20

**Authors:** Balakumaran Chandrasekar, Alan Wanke, Stephan Wawra, Pia Saake, Lisa Mahdi, Nyasha Charura, Miriam Neidert, Gereon Poschmann, Milena Malisic, Meik Thiele, Kai Stühler, Murali Dama, Markus Pauly, Alga Zuccaro

**Affiliations:** Cluster of Excellence on Plant Sciences (CEPLAS), Institute for Plant Sciences, University of Cologne, 50679 Cologne, Germany; Cluster of Excellence on Plant Sciences (CEPLAS), Institute for Plant Sciences, University of Cologne, 50679 Cologne, Germany; Max Planck Institute for Plant Breeding Research, 50829 Cologne, Germany; Cluster of Excellence on Plant Sciences (CEPLAS), Institute for Plant Sciences, University of Cologne, 50679 Cologne, Germany; Cluster of Excellence on Plant Sciences (CEPLAS), Institute for Plant Sciences, University of Cologne, 50679 Cologne, Germany; Cluster of Excellence on Plant Sciences (CEPLAS), Institute for Plant Sciences, University of Cologne, 50679 Cologne, Germany; Max Planck Institute for Plant Breeding Research, 50829 Cologne, Germany; Cluster of Excellence on Plant Sciences (CEPLAS), Institute for Plant Sciences, University of Cologne, 50679 Cologne, Germany; Cluster of Excellence on Plant Sciences (CEPLAS), Institute for Plant Sciences, University of Cologne, 50679 Cologne, Germany; Institute of Molecular Medicine, Proteome Research, University Hospital and Medical Faculty, Heinrich-Heine University Düsseldorf, Universitätsstraße 1, 40225 Düsseldorf, Germany; Cluster of Excellence on Plant Sciences (CEPLAS), Institute for Plant Sciences, University of Cologne, 50679 Cologne, Germany; Cluster of Excellence on Plant Sciences (CEPLAS), Institute for Plant Sciences, University of Cologne, 50679 Cologne, Germany; Molecular Proteomics Laboratory, Biomedical Research Centre (BMFZ), Heinrich-Heine University Düsseldorf, Universitätsstraße 1, 40225 Düsseldorf, Germany; Cluster of Excellence on Plant Sciences (CEPLAS), Institute for Plant Sciences, University of Cologne, 50679 Cologne, Germany; Institute of Plant Cell Biology and Biotechnology, Heinrich Heine University, 40225 Düsseldorf, Germany; Cluster of Excellence on Plant Sciences (CEPLAS), Institute for Plant Sciences, University of Cologne, 50679 Cologne, Germany

## Abstract

Plant pathogenic and beneficial fungi have evolved several strategies to evade immunity and cope with host-derived hydrolytic enzymes and oxidative stress in the apoplast, the extracellular space of plant tissues. Fungal hyphae are surrounded by an inner insoluble cell wall layer and an outer soluble extracellular polysaccharide (EPS) matrix. Here, we show by proteomics and glycomics that these two layers have distinct protein and carbohydrate signatures, and hence likely have different biological functions. The barley (*Hordeum vulgare*) β-1,3-endoglucanase *Hv*BGLUII, which belongs to the widely distributed apoplastic glycoside hydrolase 17 family (GH17), releases a conserved β-1,3;1,6-glucan decasaccharide (β-GD) from the EPS matrices of fungi with different lifestyles and taxonomic positions. This low molecular weight β-GD does not activate plant immunity, is resilient to further enzymatic hydrolysis by β-1,3-endoglucanases due to the presence of three β-1,6-linked glucose branches and can scavenge reactive oxygen species. Exogenous application of β-GD leads to enhanced fungal colonization in barley, confirming its role in the fungal counter-defensive strategy to subvert host immunity. Our data highlight the hitherto undescribed capacity of this often-overlooked EPS matrix from plant-associated fungi to act as an outer protective barrier important for fungal accommodation within the hostile environment at the apoplastic plant–microbe interface.

## Teaser

A β-1,3;1,6-glucan decasaccharide released from the fungal matrix by an apoplastic host hydrolase contributes to plant immune suppression and fungal accommodation.

IN A NUTSHELL **Background:** Plants secrete various hydrolytic enzymes into the apoplastic space to protect themselves against invading microbes. Some of these enzymes target the fungal cell wall polymer chitin. This enzymatic attack leads to the release of chitin oligomers, which can be perceived by the plant immune system, informing the plant to activate its defense machinery. However, chitin accounts for only a small part of most fungal cell walls. Recent studies have highlighted a largely uncharacterized, β-glucan-rich extracellular polysaccharide matrix (EPS) surrounding the cell wall of various plant-colonizing fungi.

**Question:** This EPS matrix is made of glucose and abundantly produced during colonization. As its secretion into the extracellular environment is costly for the fungus, we explored how this EPS matrix affects plant immunity and fungal colonization.

**Findings:** We demonstrated that EPS matrices from a symbiotic and pathogenic plant-colonizing fungus are distinct from the nonsoluble fungal cell walls with respect to their protein and carbohydrate composition. Enzymatic digests revealed that a secreted plant hydrolase from barley (*Hv*BGLUII) acts on these EPS matrices and releases a highly branched β-glucan decasaccharide (β-GD) fragment. This fragment is not perceived by the plant immune system but instead detoxifies reactive oxygen species produced by the plant host as a defense mechanism and contributes to host colonization. We thus have shown that the outermost fungal EPS layer represents a protective shield against oxidative stress.

**Next steps:** The diversity of linkage types and branching patterns of β-glucans not only accounts for their different biochemical properties, but also makes them important messengers for the plant, potentially encoding specific information on the approaching fungal invader. Future studies should aim to identify other plant hydrolases and the elusive glucan receptors, to disentangle the contribution of β-glucans to the communication between plant hosts and fungi.

## Introduction

The fungal cell wall (CW) consists of repeatedly branched glycan polymers and proteins that adjust according to cell type, environmental conditions, and lifestyle phases (Geoghegan et al., 2017; Gow et al., 2017). The CW of animal pathogenic fungi is commonly surrounded by a soluble gel-like extracellular polysaccharide (EPS) matrix that can contain β-glucans, a heterogeneous group of glucose polymers (Gravelat et al., 2013; Gow et al., 2017; Kang et al., 2018). Commonly, fungal β-glucans have a structure comprising a main chain of β-1,3 and/or β-1,4-glucopyranosyl units, decorated by side-chains with various branches and lengths (Han et al., 2020). The immunomodulatory properties of β-glucans derived from animal pathogens have been long recognized (Goodridge et al., 2009). β-glucans exhibit a broad spectrum of biological activities and have a dual role with respect to host immunity that depends on their chemo-physical characteristics. On the one hand, β-glucans are important microbe-associated molecular patterns (MAMPs) that are detected upon fungal colonization to trigger host immune responses in both vertebrates and invertebrates (Brown and Gordon, 2005). On the other hand, certain soluble β-glucans do not possess high immunogenic properties but are implicated in antioxidant activities and scavenging of reactive oxygen species (ROS; Han et al., 2020). Their immunological or antioxidant properties are rather complex and could be influenced by modifications in their structural characteristics such as molecular weight, substitution pattern, solubility, polymer charge, and conformation in solution (Han et al., 2020).

In plant–fungal interactions, carbohydrate metabolic processes mediated by carbohydrate-active enzymes (CAZymes) in the apoplast play a crucial role. Surface-exposed and accessible fungal polysaccharides are hydrolyzed by apoplastic CAZymes, such as chitinases and glucanases and the resulting oligosaccharides can act as elicitors to trigger a plant immune response known as pattern-triggered immunity (Silipo et al., 2010; Rovenich et al., 2016; Van Holle and Van Damme, 2018; Wanke et al., 2020, 2021; Buscaill and van der Hoorn, 2021; Ngou et al., 2021; Rebaque et al., 2021; Yuan et al., 2021). Recently, we described a soluble extracellular β-glucan matrix produced by endophytic fungi during root colonization of *Arabidopsis thaliana* (hereafter Arabidopsis) and *Hordeum vulgare* (hereafter barley; Wawra et al., 2019). Little is known about the biochemical properties, composition, and function of this EPS matrix, but its detection in beneficial and pathogenic fungi strongly suggests a conserved role in counteracting environmental and immunological challenges during fungal growth and plant colonization (El Oirdi et al., 2011; Mahdi et al., 2021; Wanke et al., 2021). It is therefore crucial to investigate the structure and function of these soluble β-glucans and the hydrolytic events mediated by host apoplastic CAZymes during plant–fungal interactions. In this study, we have characterized the CW and the soluble EPS matrix produced by two distantly related fungi, the beneficial root endophyte *Serendipita indica* (Basidiomycota), and the pathogenic fungus *Bipolaris sorokiniana* (Ascomycota), separated by over 649 million years of evolution (Taylor and Berbee, 2006; Lutzoni et al., 2018). Proteomics and glycomics revealed that β-glucan-binding proteins with cell wall integrity and stress response component (WSC) domains and β-1,3;1,6-glucan polysaccharides are enriched in the soluble EPS matrix compared to the CW layer. Treatment of the fungal EPS matrices with the apoplastic barley β-1,3-endoglucanase *Hv*BGLUII released a β-1,3;1,6-glucan decasaccharide (β-GD) with a mass/charge (m/z) of 1661 Da. Proton nuclear magnetic resonance (^1^H NMR) of β-GD is consistent with a heptameric β-1,3-glucan backbone substituted with three monomeric β-glucosyl residues at O-6. The β-GD is resilient to further enzymatic digestion by glycoside hydrolase 17 (GH17) family members and is immunologically inactive in a β-1,6-glucan side-branch-dependent manner. This low molecular weight soluble β-GD is able to efficiently scavenge ROS and to enhance colonization, corroborating its role as a previously undescribed fungal carbohydrate-class effector. The release of a conserved β-GD from the β-glucan-rich EPS matrix of a beneficial and a pathogenic fungus indicates that the utilization of this outermost soluble polysaccharide layer as a protective shield against oxidative stress and ROS-mediated host signaling is a common fungal strategy to withstand apoplastic defense responses during plant colonization.

## Results

### Beneficial and pathogenic fungi produce a gel-like β-glucan EPS matrix surrounding their CWs

**Figure 1:**
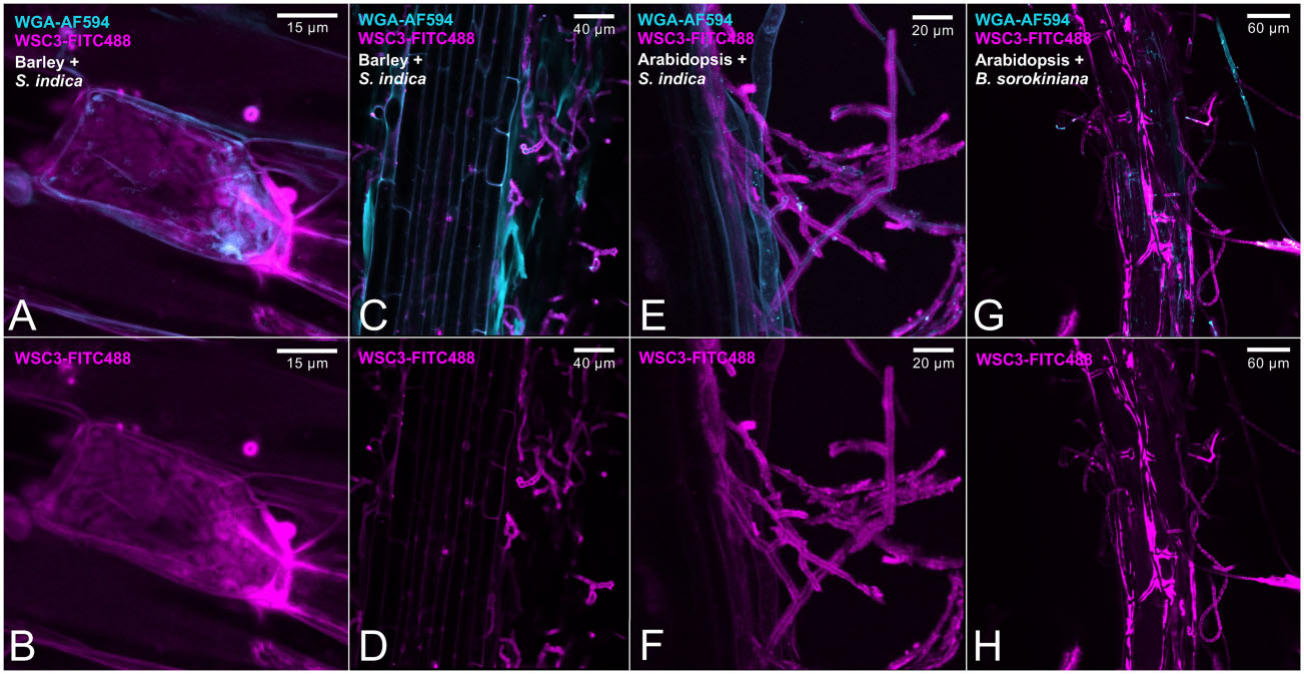
Fungal EPS matrix revealed by the fluorescently labeled β-glucan binding lectin *Si*WSC3-His-FITC488 during root colonization. The β-glucan-binding *Si*WSC3-His and the chitin-binding WGA lectins were used as molecular probes to visualize the fungal EPS matrix and CW of *S. indica* and *B. sorokiniana*, respectively. Magenta pseudocolor corresponds to FITC488-labeled *Si*WSC3-His. Cyan pseudocolor corresponds to WGA-AF594. (A), (C), (E), and (G) are merged confocal microscopy images of *Si*WSC3-His-FITC488 and WGA-AF594. (B), (D), (F), and (H) display the EPS matrix of *S. indica* or *B. sorokiniana* stained by *Si*WSC3-His-FITC488 during colonization of Arabidopsis or barley roots. (A) and (B) show *S. indica* intracellular colonization of a barley root cell with abundant production of the β-glucan EPS matrix. The microscopy was repeated at least 10 times with two independent *Si*WSC3-His-FITC488 batches and independent Arabidopsis or barley plants colonized by *S. indica* or *B. sorokiniana*. The fungal matrix was not a sporadic observation but regularly observed with both fungi. WGA, wheat germ agglutinin.

We recently reported on a gel-like EPS matrix surrounding the hyphae of different fungi during colonization of plant hosts (Wawra et al., 2019; Wanke et al., 2021), suggesting that the secretion of soluble glycans is a common feature of plant‐associated fungi independent of their lifestyle and taxonomy. This finding motivated us to investigate the biochemical characteristics, composition, and function of this matrix in the beneficial root endophyte *S. indica* and in the pathogenic fungus *B. sorokiniana*. To this end, we labeled the β-1,3-glucan-binding lectin PIIN_05825 (*Si*WSC3-His-FITC488) from *S. indica* by applying an improved FITC488 conjugation protocol (see “Materials and methods”) and used it as a molecular probe for localization studies of the fungal EPS matrix in planta. *Si*WSC3-His-FITC488 signal accumulated around the wheat germ agglutinin-stained chitin layer in both fungi (Figure 1), strongly indicating that β-1,3-glucans are abundant in the outer EPS matrix. This expands the repertoire of fungal β-glucan-binding lectins that can be used as molecular probes to fluorescently label the fungal EPS matrix during live cell imaging (Wawra et al., 2016).

### The *S. indica* EPS matrix, CW, and culture filtrate represent three functionally distinct but interconnected compartments

**Figure 2:**
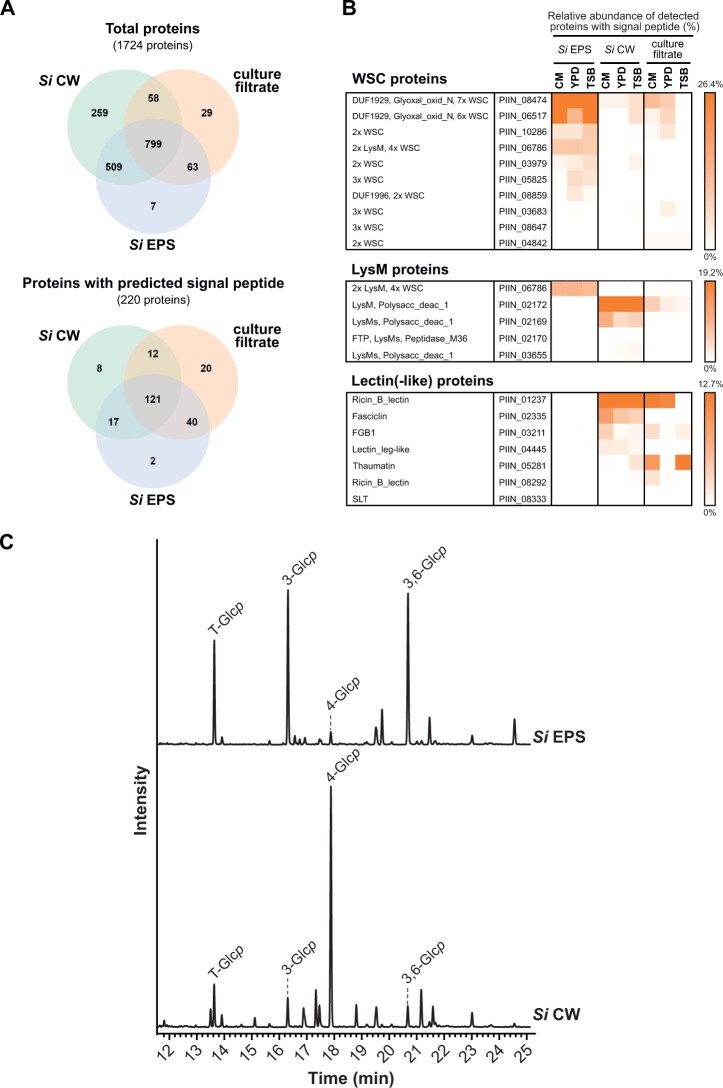
Proteomics and glycosyl linkage analysis of *S. indica* EPS matrix and CW. A, Venn diagram of proteins identified in the EPS matrix, CW, and/or culture filtrate from three cultivation media (CM, YPD, and TSB). B, Proteins with WSC domain/s show higher abundances in the EPS matrix of *S. indica* (see also Supplemental Figure S2 and Supplemental Data Sets S1, S2, and S3). The relative abundance of each protein was calculated using LFQ intensity values and is depicted in percentage. C, Glycosidic linkage analysis of *S. indica* EPS and CW preparations. 3-glucose and 3,6-glucose are abundant in the EPS matrix, whereas 4-glucose is abundant in the CW of *S. indica.* The experiment was performed with four independent biological replicates of *Si* EPS and *Si* CW and the TIC of one of the replicates is represented. TIC, total ion chromatogram; LysM, lysine motif; *p*, pyranose; *Si*, *Serendipita indica*.

To identify secreted proteins associated with the EPS matrix, the CW, and/or the culture filtrate, we performed quantitative proteomics with protein extracts from these three compartments from *S. indica* axenically grown in three different media (complete medium [CM], yeast-extract peptone dextrose [YPD], tryptic soy broth [TSB]; Supplemental Figure S1). We identified 1,724 proteins from all media and compartments (Supplemental Data Set S1; Figure 2A). Among those, 220 proteins carrying a predicted signal peptide were further analyzed for their domain architecture using the Pfam database (Supplemental Data Set S2; Figure 2A). Glucan-binding proteins with at least one WSC domain were more abundant or uniquely present in the EPS matrix compared to the CW (enrichment score 0.96, Benjamini–Hochberg corrected *P* = 0.00003) or the culture filtrate (enrichment score 0.6, Benjamini–Hochberg corrected *P* = 0.02), irrespective of the medium used (Figure 2B; Supplemental Figure S2 and Supplemental Data Set S3). Four of them, including *Si*WSC3 (PIIN_08474, PIIN_06786, PIIN_03979, and PIIN_05825), were among the most abundant proteins consistently found in this compartment (Figure 2B; Supplemental Figure S2), thereby confirming our in planta localization study (Figure 1). Gene expression analyses revealed an induction of these genes during *S. indica* colonization of barley and Arabidopsis over time (Wawra et al., 2019; Supplemental Figure S3). Additionally, several *S. indica* proteases and CAZymes were more abundant in the EPS matrix and culture filtrate compared to the CW (Supplemental Figure S2 and Supplemental Data Set S3), suggesting that the matrix may serve as a transient storage depot for these enzymes. In the CW fraction, we identified two chitin-binding LysM proteins (PIIN_02172 and PIIN_02169) and several other lectin-like proteins, including the β-1,6-glucan-binding effector *Si*FGB1 (Wawra et al., 2016; PIIN_03211) and the ricin B lectin (PIIN_01237). Most of these lectins were also present in the culture filtrate, indicating that besides their ability to bind to CW components, such as chitin and glucan, they also function as soluble lectins in the extracellular environment (Figure 2B; Supplemental Figure S2). This is in agreement with the function of *Si*FGB1, which has the potential to alter fungal CW composition and properties as well as suppress β-glucan-triggered immunity in the apoplast of different plant hosts (Wawra et al., 2016). Proteins containing cellulose-binding CBM_1 or starch-binding CBM_20 domains were found at higher abundances in the culture filtrate (Supplemental Figure S2). Based on the distribution and nature of the carbohydrate-binding proteins, we propose that the EPS matrix, the CW, and the culture filtrate represent three functionally distinct but interconnected compartments.

### *Serendipita indica* EPS matrix and CW have different sugar compositions

The different amount of glycan-binding proteins within the EPS matrix and CW layer prompted us to investigate the glycan composition of these two compartments. Protein-free EPS matrix and CW preparations from *S. indica* grown in TSB medium were subjected to glycosyl linkage analysis for neutral sugars (Ciucanu, 2006; Liu et al., 2015). About 85% of the detected glycosidic linkages could be annotated based on the retention times and the mass spectra profile of the sugar residues (Supplemental Data Sets S4 and S5). Terminal glucose, 3-glucose, and 3,6-glucose were the dominant glycosidic linkages (∼35%) observed in the EPS matrix compared to other glycosidic sugar residues (Figure 2C; Supplemental Figure S4 and Supplemental Data Set S4). In contrast, in the CW fraction, 4-linked glucose was more abundant (∼45%, Figure 2C; Supplemental Figure S4 and Supplemental Data Set S5) than in the EPS matrix. Further analyses are required to clarify the type of glycosidic linkages that can be α-, β-, or mixed type.

**Figure 3:**
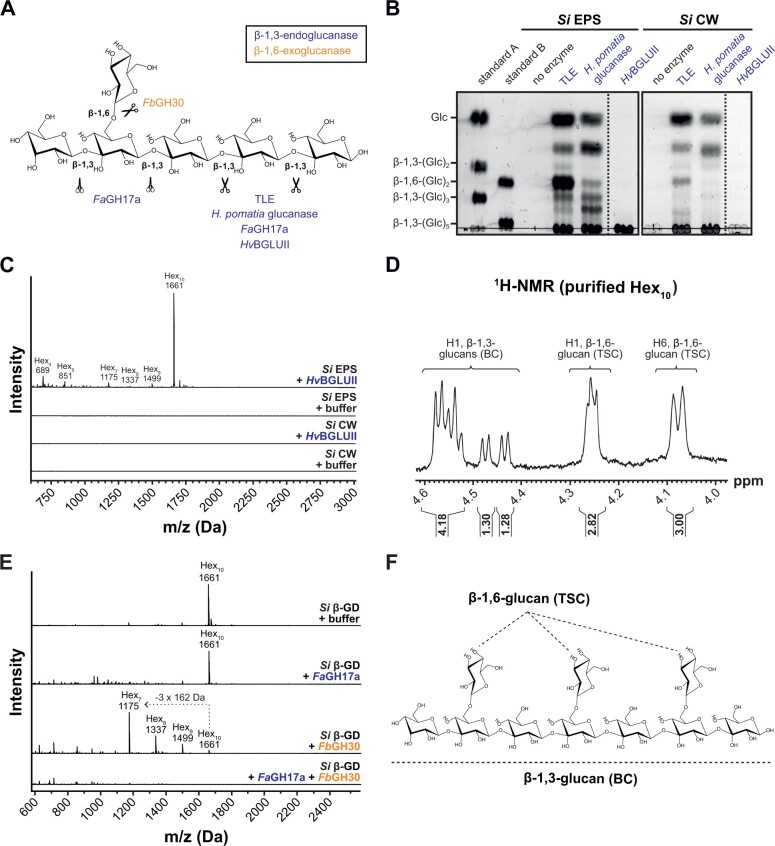
The β-1,3;1,6-glucan decasaccharide β-GD is released from the *S. indica* EPS matrix upon treatment with the barley apoplastic glycosyl hydrolase *Hv*BGLUII. A, Glycosyl hydrolases specific for β-1,3;1,6-glucans were used for the characterization of the EPS matrix, CW, and β-GD. The β-1,3-endoglucanases from *T. harzianum* (TLE) and *H. pomatia* as well as *Fa*GH17a and *Hv*BGLUII are shown as open scissors (in blue). *Fa*GH17a is represented as closed scissors because it does not hydrolyze glycosidic bonds of β-1,3-glucosyl residues substituted with β-1,6-glucosyl residues (in blue). *Fb*GH30 is a β-1,6-exoglucanase (in orange). B, Analysis of digested EPS matrix or CW fractions by TLC. Several glucan fragments with different lengths are released from the EPS matrix and CW by the action of TLE and *H. pomatia* β-1,3-glucanase. *Hv*BGLUII releases a glucan fraction from the β-glucan-containing EPS matrix but not from the CW. The experiment was repeated twice with *Si* EPS and *Si* CW isolated under different medium conditions (YPD and CM) and similar results were obtained. C, Analysis of digested EPS or CW fractions by MALDI-TOF mass spectrometry. The 1,661 Da β-GD corresponding to 10 hexoses is released from the EPS matrix but not from the CW of *S. indica*. The representative DP of hexoses is indicated on top of the *m/z* (M+Na)^+^ masses of oligosaccharides. The digestion of *Si* EPS with *Hv*BGLUII was repeated independently more than three times with a similar result and the digestion of *Si* CW with *Hv*BGLUII was performed two times with a similar result. D, ^1^H NMR spectrum of HPLC purified β-GD. E, Treatment of β-GD with various hydrolases followed by MALDI-TOF analysis of the products. The loss of three hexoses (−3 × 162 Da) as a result of treatment with *Fb*GH30 is indicated with a dotted arrow. The experiment was performed two times with a similar result. F, Structure of the β-GD based on the ^1^H NMR spectrum. β-GD consists of a linear β-1,3-glucan backbone substituted with β-1,6-glucosyl moieties. *Si*, *Serendipita indica*; DP, degree of polymerization; Hex_n_, oligosaccharides with the indicated hexose composition; BC, backbone chain; TSC, terminal side-chain.

Next, we treated the EPS matrix and CW with β-glucanases from *T. harzianum* (TLE) and *H. pomatia* and analyzed the digested products by thin-layer chromatography (TLC) and matrix-assisted laser desorption ionization time-of-flight mass (MALDI-TOF; Figure 3A). Several glucan fragments with various degrees of polymerization could be detected in the digested fraction confirming that β-glucans are present in both layers (Figure 3B; Supplemental Figure S5). Altogether, these data demonstrate that the EPS matrix and the CW of *S. indica* display major differences in the linkage compositions of their neutral sugars.

### A β-glucan decasaccharide is released from the *S. indica* EPS matrix by a barley glucanase

We recently reported that several β-glucanases belonging to the GH17 family accumulate in the apoplast of barley roots during colonization by *S. indica* (Wawra et al., 2016). Among them, the β-glucanase *Hv*BGLUII (P15737) was consistently found at different colonization stages but also in mock-treated plants (Wawra et al., 2016; Supplemental Data Set S6), suggesting that this may be an ubiquitous apoplastic enzyme in root tissues. To investigate the activity of *Hv*BGLUII on the fungal CW and/or on the EPS matrix, we analyzed the digested fraction by TLC analysis after enzymatic incubation. Treatment of the EPS matrix with *Hv*BGLUII led to the release of a glucan fraction found at the sample origin spot that could not be further resolved under the TLC separation conditions used (Figure 3B). The corresponding band was not detected in the CW digestion (Figure 3B), indicating that *Hv*BGLUII is only active on the EPS matrix. Other β-1,3-glucanases (TLE and *H. pomatia* β-1,3-glucanase) were able to release oligosaccharides from both EPS and CW preparations, indicating that both preparations contain β-glucans that are enzyme accessible (Figure 3B; Supplemental Figure S5).

To further characterize the structure of the compound released by *Hv*BGLUII from the EPS matrix, we performed MALDI-TOF and glycosyl linkage analyses. An oligosaccharide with a *m/z* of 1,661 Da, corresponding to 10 hexoses (referred to as β-glucan decasaccharide [β-GD] fragment) with 3- and 3,6-linked glucoses was detected in high abundance compared to other oligosaccharides with various degrees of polymerization (DP4–DP9, Figure 3C; Supplemental Figure S6A). Neither the β-GD nor the other oligosaccharides were detected in the supernatant of the digested CW preparation (Figure 3C), confirming the TLC result (Figure 3B). Furthermore, the β-GD was also released from the EPS matrix of *S. indica* grown in CM medium (Supplemental Figure S6B), suggesting that the growth conditions do not notably influence the release of the β-GD.

To further asses the structure of β-GD, the fragment was purified using reverse-phase chromatography (Supplemental Figure S7) and subjected to ^1^H NMR spectroscopy (Figure 3D). ^1^H NMR analysis displayed characteristic proton signals for β-1,3;1,6-glucan (Kim et al., 2000; Tada et al., 2009; Lowman et al., 2011). Since β-GD was reduced prior to the NMR analysis, only nine anomeric carbohydrate signals were present. The anomeric ^1^H NMR signals of the six internal β-1,3-glucan backbone moieties were identified at 4.4–4.6 ppm (Figure 3D). They appear as a multiplet due to overlapping proton doublet signals at 4.5–4.6 ppm (four protons), a doublet at 4.48 ppm (J 7.8 Hz, one proton) representing the second glucose unit next to the reducing end and a doublet at 4.44 ppm (J 7.8 Hz, one proton) representing the non-reducing end of the oligosaccharide backbone. Anomeric NMR signals were also observed for the β-1,6-D-side-chain substituents at 4.26 ppm (three protons), indicating that the oligosaccharide contains three individual monomeric substituents. This is confirmed by the H6 NMR signal of β-1,6-D-glucose substituents at 4.08 ppm (three protons). In conclusion, the ^1^H NMR analysis indicates that β-GD consists of seven β-1,3-linked D-glucose backbone units substituted with three terminal β-1,6-glucose units. The order of the substituents on the backbone could not be established by the NMR analysis performed here.

To validate the ^1^H NMR results, we took advantage of the two well-characterized glycosyl hydrolases, *Fa*GH17a and *Fb*GH30 (Becker et al., 2017; Wanke et al., 2020). *Fa*GH17a is an endoglucanase specifically active on unsubstituted β-1,3-glucans and *Fb*GH30 is an exoglucanase specific for β-1,6-glycosidic linkages (Figure 3A). The β-GD was treated with *Fa*GH17a or *Fb*GH30 or a combination of the two enzymes and the digested samples were analyzed using MALDI-TOF mass spectrometry (Figure 3E). Digestion with *Fb*GH30 resulted in ion signals that represent the enzymatic removal of one (*m/z* 1,499), two (*m/z* 1,337), or most pronounced three glucosyl moieties (m/z 1,175), confirming the presence of three β-1,6-glucose units in β-GD. Digestion with *Fa*GH17a alone did not alter the molecular weight of β-GD indicating that potential enzyme hydrolysis sites are blocked by its side-chain substituents. In contrast, the combined treatment with both enzymes led to complete hydrolysis of the β-GD (Figure 3E). Taken together, these results demonstrate that the β-GD released from the EPS matrix by the action of *Hv*BGLUII is a decasaccharide with seven β-1,3-glucosyl units substituted with three β-1,6-glucosyl units as depicted in Figure 3F.

### The apoplastic *Hv*BGLUII fosters MAMP-triggered immunity that is counteracted by the uncleavable β-GD

**Figure 4:**
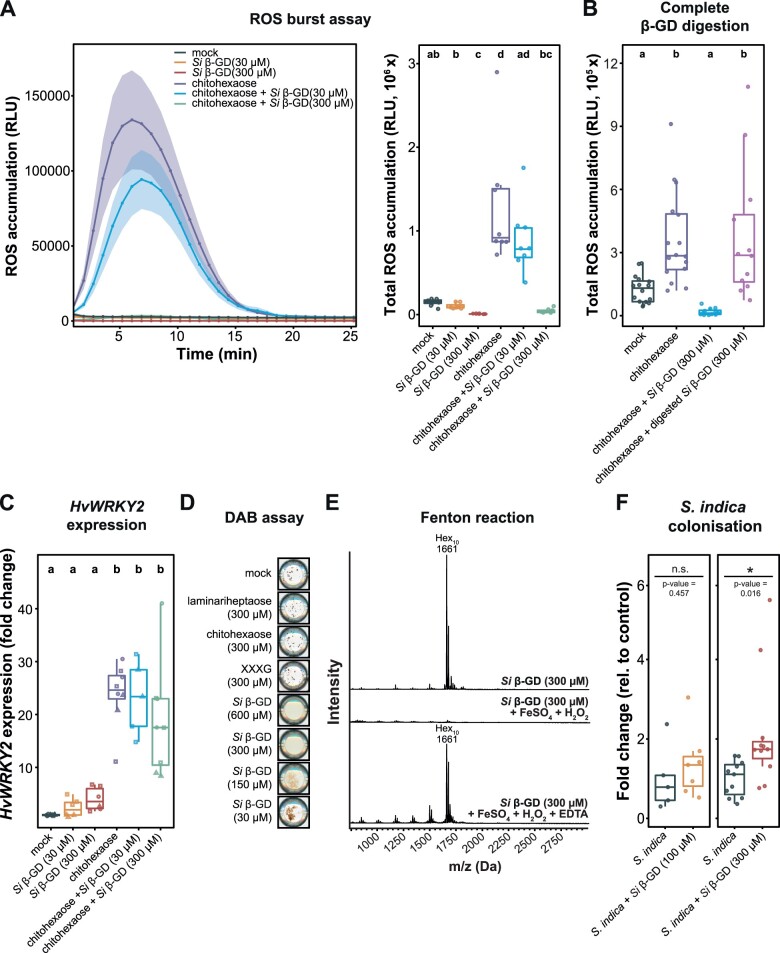
The β-GD released from *S. indica* EPS matrix scavenges ROS and enhances host colonization. A, Apoplastic ROS accumulation after treatment of barley roots of 8-day-old plants with 25-µM chitohexaose and/or purified β-GD from *S. indica.* ROS accumulation was monitored via a luminol‐based chemiluminescence assay. Treatment with Milli-Q water was used as mock control. Boxplot represents total ROS accumulation over the measured time period. Values represent mean ± sem from eight wells, each containing four root pieces. In total, roots from 16 individual barley plants were used per experiment. The assay was performed at least four times with independent β-GD preparations. Letters represent statistically significant differences in expression based on a one-way ANOVA and Tukey’s post hoc test (significance threshold: *P* ≤0.05). B, Prior to treatment of barley root pieces with the elicitors, β-GD was digested overnight (25°C, 500 rpm in heat block) with the glucanases *Fa*GH17a and *Fb*GH30, which led to complete digestion of β-GD (see also Figure 3E). As control, β-GD without the addition of enzymes (but instead with an equal volume of Milli-Q water) was treated similarly. Barley root pieces were treated with Milli-Q water (*n* = 16) and 25-µM **Figure 4** (continued) chitohexaose alone (*n* = 16) or in combination with digested or undigested β-GD (300 µM, *n* = 12). The experiment was performed twice with similar results. Statistically significant differences are indicated by different letters based on a one-way ANOVA and Tukey’s post hoc test (significance threshold: *P* ≤0.05). C, Barley root pieces were collected 1 h after elicitor treatment and further processed for RNA extraction and cDNA synthesis. Expression changes of the elicitor-responsive gene *HvWRKY2* were analyzed by RT-qPCR. Fold change expression were calculated by normalization to housekeeping gene expression (*HvUBI*) and mock treatment. Data from three independent experiments are indicated by different dot shapes. Letters represent statistically significant differences in expression based on two-way ANOVA (additive model, treatment + experiment) and Tukey’s post hoc test (significance threshold: *P* ≤0.05). Significant differences were associated with different treatments (*F* = 11.629, *P* = 1.58 × 10^−6^), but not with independent experiments (*F* = 2.227, *P* = 0.124). D, The capability of different carbohydrates to prevent hydrogen peroxide-based and horseradish peroxidase-catalyzed oxidation and precipitation of DAB was monitored. Respective sugars (or Milli-Q water as mock control) were pre-incubated with 1-mM H_2_O_2_ and 0.05-µM horseradish peroxidase before DAB (50 µM) was added. Scans of wells from 96-well plates were performed 16 h after DAB addition. The experiment was performed three times with similar results. E, Oxidative degradation of *S. indica* EPS matrix-derived β-GD (300 µM) by H_2_O_2_ was detected with an overnight Fenton reaction (1-mM H_2_O_2_, 100-µM FeSO_4_) followed by MALDI-TOF mass spectroscopic analysis. As controls, either sugar alone or the samples supplemented with 100-µM EDTA were used. F, Colonization of barley roots by *S. indica* upon daily application of sterile Milli-Q water (mock) or β-GD (100 or 300 µM). Fungal colonization in each biological replicate was assessed by RT-qPCR comparing the expression of the fungal housekeeping gene *SiTEF* and the plant gene *HvUBI* (*n* = 5–11). Boxplot elements in this figure: center line, median; box limits, upper and lower quartiles; whiskers, 1.5× interquartile range. Statistical significance was determined on the nontransformed values (before normalization to *S. indica* control treatment) using a two-tailed Student’s *t* test (*P ≤0.05). *Si*, *Serendipita indica*; RLU, relative light units; XXXG, xyloglucan heptasaccharide.

Since β-glucans represent an important class of microbial cell surface glycans able to trigger plant immune responses (Fesel and Zuccaro, 2016; Wanke et al., 2020), we performed ROS burst assays with *S. indica* CW and EPS matrix as well as with the enzymatically released β-GD to test their immunogenic potential on barley roots. Whereas the application of the fungal MAMP chitohexaose (positive control) triggered apoplastic ROS accumulation, incubation with *S. indica* CW and EPS matrix only marginally induced ROS accumulation (Supplemental Figure S8). Applications of the β-GD led to significantly lower ROS levels compared to the mock treatment (Figure 4A; Supplemental Figure S9). This prompted us to test the ability of this fragment to affect ROS levels during co-treatment with different MAMPs (Figure 4A; Supplemental Figures S10 and S11). The combined application of chitohexaose and β-GD led to a decreased accumulation of ROS with increasing concentrations of added β-GD (Figure 4A; Supplemental Figures S9 and S10). Combined digestion of the β-GD with the endoglucanases *Fa*GH17a and *Fb*GH30 restored the chitohexaose-triggered ROS burst (Figure 4B), thereby highlighting that the decreased accumulation of apoplastic ROS is linked to the presence of an—at least largely—intact β-GD. Impaired ROS accumulation was not observed with *S. indica* CW or EPS matrix preparations (Figure S8). Additionally, the application of the β-GD significantly reduced apoplastic ROS accumulation in barley roots treated with the β-1,3;1,6-glucan laminarin and in Arabidopsis seedlings treated with the flagellin-derived peptide flg22 (Supplemental Figure S11), substantiating a general function of the β-GD irrespective of plant species or elicitor.

To clarify whether the β-GD interferes with the MAMP perception machinery or detoxifies ROS, we tested its effect on further early and late immune responses triggered by chitohexaose. In barley, *HvWRKY2* has been demonstrated to act as a reliable marker for the onset of early immune responses among a wide range of elicitors applied to barley (Shen et al., 2007; Liu et al., 2014; Wanke et al., 2020). Despite the reduction of the oxidative burst, chitohexaose-triggered *HvWRKY2* expression was not reduced by the application of the β-GD (Figure 4C; Supplemental Figure S10C). This shows that the β-GD does not prevent MAMP perception but acts on the released ROS. Furthermore, treatment with the β-GD alone did not lead to a significant increase in *HvWRKY2* expression, supporting the notion that this β-glucan fragment does not exhibit an immunogenic activity in barley roots. In support of this, treatments of Arabidopsis seedlings with β-GD did not induce rapid intracellular calcium fluxes (Supplemental Figure S12), an early hallmark of plant immune responses (Boller and Felix, 2009). These results are surprising due to the structural similarity of β-GD to laminarihexaose and laminarin, two potent ROS elicitors in different plant species, including barley and Arabidopsis (Wanke et al., 2020). Thus, the frequency and position of β-1,6-glucose substituents may define both their immunomodulatory potential as MAMP as well as their biochemical activity as ROS scavengers. To confirm this hypothesis, we treated laminariheptaose, the β-1,3-backbone of β-GD, with *Hv*BGLUII for 1 h or overnight and tested those preparations in ROS burst assays. Barley BGLUII was capable of digesting the laminariheptaose to glucose and laminaribiose (Supplemental Figure S13). The activity of *Hv*BGLUII on the laminariheptaose led to higher ROS accumulation compared to incubation with undigested laminariheptaose (Supplemental Figure S13). This demonstrates that *Hv*BGLUII is a host defense enzyme that releases potent MAMPs from unbranched β-1,3-glucan oligomers likely derived from the fungus (Figure 2C), playing a role in host glycan perception.

### The β-GD scavenges apoplastic ROS

Numerous studies have highlighted the capability of sugars and specifically of β-glucans to act as ROS scavengers, contributing to the intracellular antioxidant system in different eukaryotes (Benaroudj et al., 2001; Nishizawa et al., 2008; Valluru and Van den Ende, 2008; Peshev et al., 2013; Lei et al., 2015; Boulos and Nystrom, 2017). To test whether the β-GD can directly act as an antioxidant, we performed an in vitro 3,3′-diaminobenzidine (DAB) assay. In the presence of hydrogen peroxide and horseradish peroxidase as catalyst, DAB is oxidized and polymerizes, ultimately leading to the formation of a brown, water-insoluble precipitate (Figure 4D). At low concentrations of β-GD (30–150 µM), DAB still oxidizes but forms less precipitates compared to the mock control. Formation of precipitates was completely inhibited at higher concentrations of β-GD (300–600 µM; Figure 4D). The inhibition of DAB precipitation can be explained by the ability of β-GD to scavenge ROS in a concentration dependent manner. Other CW-associated sugars such as chitohexaose, laminariheptaose, and a xyloglucan heptasaccharide (XXXG) did not interfere with DAB precipitation (Figure 4D). Mechanistically, nonenzymatic scavenging of hydroxyl radicals by sugars is based on their oxidation, which can lead to cleavage of glycosidic linkages and the formation of less reactive sugar radicals that further cross-react with themselves or other sugars (Matros et al., 2015). To validate if the oxidation of the decasaccharide contributes to ROS scavenging, we performed a Fenton reaction-based assay with the β-GD followed by MALDI-TOF analysis. In the presence of the Fenton reagents, the peak at 1,661 Da, corresponding to the β-GD, was no longer detected, suggesting that the fragment might have undergone oxidative degradation and/or have changed its chemo-physical properties by the activity of hydroxyl radicals produced by the Fenton reaction (Figure 4E). The oxidative degradation of the β-GD could be rescued in the presence of ethylenediaminetetraacetic acid (EDTA), which chelates the catalytic iron involved in the formation of hydroxyl radicals. Complete degradation was not observed for the structurally related laminariheptaose, chitohexaose, and XXXG (Supplemental Figure S14). Altogether, these results demonstrate that the activity of *Hv*BGLUII on the *S. indica* EPS matrix does not release a MAMP that initiates plant defense responses, but a fragment that can detoxify apoplastic ROS possibly via oxidative degradation. These results highlight a hitherto undescribed function of the fungal EPS matrix as a protective layer to mitigate oxidative stress during plant–microbe interaction.

To explore whether the β-GD can facilitate root colonization during early interaction, we performed colonization assays on barley roots in the presence of various β-GD concentrations (Figure 4F). The addition of β-GD slightly increased colonization at 100 µM and led to an on average 2.15-fold increase of fungal colonization at 300 µM after 3 days post inoculation (dpi). This demonstrates that an EPS matrix-resident decasaccharide that is released by a plant glucanase can act as a carbohydrate-class effector enhancing fungal colonization.

### The antioxidative properties of the β-GD are conserved among pathogenic and beneficial fungi

**Figure 5:**
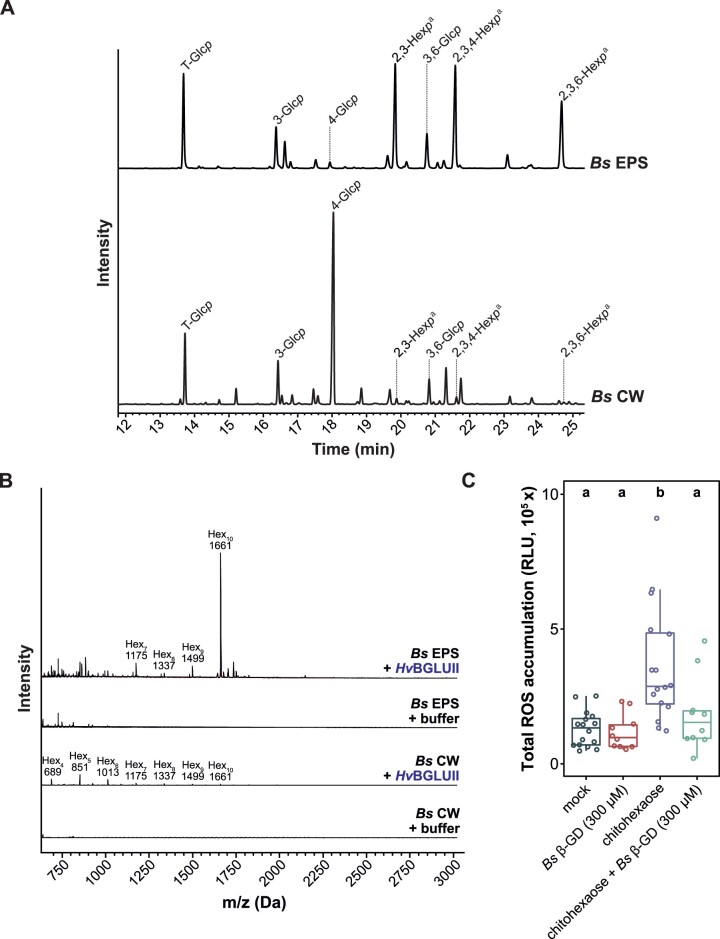
The β-GD derived from the hydrolysis of *B. sorokiniana* EPS matrix exhibits antioxidative properties. A, Glycosidic linkage analysis of *B. sorokiniana* EPS matrix and CW preparations. 2,3-hexopyranose, 2,3,4- hexopyranose, and 2,3,6- hexopyranose are abundant in the EPS matrix, whereas 4-glucose is abundant in the CW of *B. sorokiniana*. The experiment was performed with three independent biological replicates of *Bs* EPS and *Bs* CW and the TIC from one of the replicates is represented. B, Analysis of digested EPS or CW fractions by MALDI-TOF mass spectrometry. The 1,661-Da β-GD corresponding to 10 hexoses is released from the EPS matrix but not from the CW of *B. sorokiniana*. The representative DP of hexoses is indicated on top of the *m/z* (M+Na)^+^ masses of oligosaccharides. The digestion of *Bs* EPS with *Hv*BGLUII was repeated independently three times with a similar result and the digestion of *Bs* CW with *Hv*BGLUII was performed once. C, ROS burst assay was performed on barley roots treated with Milli-Q water (mock), chitohexaose (25 µM), *Bs* β-GD (300 µM), or a combination of chitohexaose and *Bs* β-GD. Boxplots represent total cumulative ROS accumulation over a measured time interval of 25 min. Each data point in the boxplot represents the integrated value from an individual well (center line, median; box limits, upper and lower quartiles; whiskers, 1.5× interquartile range). The experiment was performed three times with similar results. Statistically significant differences are indicated by different letters based on a one-way ANOVA and Tukey’s post hoc test (significance threshold: *P* ≤0.05). *Bs*, *Bipolaris sorokiniana*; DP, degree of polymerization; *p*, pyranose. ^a^exact sugar moiety unknown; overrepresentation of linkages due to undermethylation cannot be excluded.

To clarify whether the observed properties of the β-GD are conserved among fungi with different lifestyles and taxonomic positions, we performed glycosyl linkage analysis of the CW and EPS matrix of *B. sorokiniana* grown in YPD medium. As observed for *S. indica*, the relative abundance of 4-linked glucose in the *B. sorokiniana* CW was higher compared to other sugar residues (Figure 5A; Supplemental Figure S15 and Supplemental Data Set S7). In the *B. sorokiniana* EPS matrix, 3-glucose and 3,6-glucose represented only a minor fraction (∼10%) compared to 2,3-hexose, 2,3,4-hexose, and 2,3,6-hexose (∼40%) and this is different from the EPS matrix of *S. indica* (Figure 5A; Supplemental Figure S15 and Supplemental Data Set S8). However, the presence of 3- and 3,6-linked glucose suggests that β-glucans with similar structures to *S. indica* glucans are also present in the EPS matrix of *B. sorokiniana*. Indeed several glucan fragments with various degrees of polymerization (DP3–DP10) could be release from digestion with the TLE but not from the buffer control (Supplemental Figure S16), indicating that β-glucans are present in the EPS matrix.

Since *Hv*BGLUII was detected in the apoplastic fluid of *B. sorokiniana* challenged barley roots and the gene is highly induced during *B. sorokiniana* root colonization (Supplemental Data Sets S6 and S9), we investigated whether *Hv*BGLUII can also release oligosaccharides from the *B. sorokiniana* EPS matrix. We detected a 1,661 Da fragment in high abundance representing a decasaccharide (Figure 5B). To characterize the structure of the 1,661 Da fragment from *B. sorokiniana*, we incubated it with *Fa*GH17a or *Fb*GH30 or a combination of the two enzymes. MALDI-TOF analysis revealed that this fragment has the same digestion profile and thus most likely the same structure as *S. indica* β-GD (Supplemental Figure S17), demonstrating that β-GD is conserved among distantly related fungi.

Next, to investigate the immunogenic properties of *B. sorokiniana* β-GD, we tested it in ROS burst assays using barley roots. Consistent with the data obtained from *S. indica*, the enzymatically released *B. sorokiniana* β-GD, but not crude EPS matrix or CW preparations, displayed a decrease in ROS accumulation when co-applied with chitohexaose (Figure 5C; Supplemental Figure S18). Collectively, our results indicate that the antioxidative property of the β-GD is a common feature among plant-associated fungi.

## Discussion

Fungi synthetize and secrete a wide range of glycans, which are crucial determinants of microbe–microbe and microbe–host interactions. Despite variations in glycan structures and composition of the CW and the surrounding EPS matrix between fungal species, β‐glucans are generally the most abundant and complex structural components. Their recognition by host receptors activates immune responses, such as accumulation of ROS and CAZymes in the extracellular apoplastic space, which hamper host colonization (El Oirdi et al., 2011; Wanke et al., 2020, 2021; Rebaque et al., 2021). In order to overcome or bypass such responses, fungi employ different extracellular strategies (Buscaill and van der Hoorn, 2021). Common fungal countermeasures to prevent recognition and hydrolysis of their surface exposed glycans involve converting, depleting or masking highly immunoactive CW components. Although effective, these countermeasures may not always be employed because some of these glycan structures mediate important processes that are beneficial to the fungus. This explains why some glycan structures are highly conserved and cannot be extensively modified. Thus, fungi have additionally evolved apoplastic glycan-binding effector proteins that either sequester immunoactive CW‐derived elicitors from the apoplast to prevent their recognition or shield them from hydrolysis. Additionally, fungi secrete CAZyme inhibitors and proteases that cleave host hydrolytic enzymes (Ham et al., 1997; Rovenich et al., 2016; Rocafort et al., 2020). Less is known about fungal cytoplasmic effector proteins targeting the disruption of glycan signaling inside plant cells.

**Figure 6:**
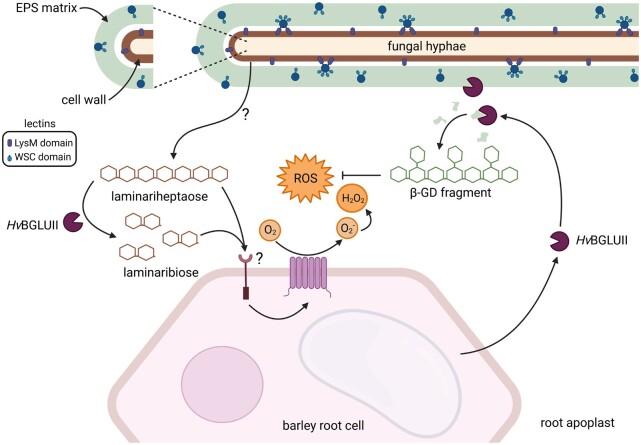
Model for the production and function of the conserved fungal EPS-derived β-1,3;1,6-glucan decasaccharide. The fungal-responsive GH17 family member *Hv*BGLUII is found in the apoplast of barley roots and acts on β-1,3-glucan. Digestion of linear β-1,3-glucan (laminariheptaose) with *Hv*BGLUII enhances ROS accumulation in barley roots, corroborating its role as a host defense enzyme with a function in β-glucan perception. To counteract the activity of *Hv*BGLUII, plant-colonizing fungi produce a β-1,3;1,6-glucan-rich EPS matrix. The activity of *Hv*BGLUII on the EPS matrix releases a conserved β-GD, which is resilient to further digestion by GH17 family members. The β-GD acts as a carbohydrate-class effector by scavenging ROS and enhancing fungal colonization. Lectins containing WSC domains are enriched in the outer EPS matrix and lectins containing LsyM domains are enriched in the CW of *S. indica*. Graphical illustration was designed with the BioRender online tool.

Here, we identified a previously undescribed extracellular fungal counterdefensive strategy to subvert host immunity that involves the hijacking of widely distributed plant apoplastic endoglucanases to release a conserved β-1,3;1,6-glucan decasaccharide with ROS scavenging properties from the extracellular polysaccharide matrix of different fungi (Figure 6). Several classes of plant proteins, called pathogenesis-related (PR) proteins, are induced in response to fungal colonization. Among these proteins the family of PR-2 proteins, which are β-1,3-endo-type glucanases, is long known (Stintzi et al., 1993; Leubner-Metzger and Meins, 1999). In seed plants, β-1,3-glucanases are widely distributed, highly regulated and abundant in the apoplast or vacuoles. Besides their role in the plant response to microbial pathogens and wounding, these enzymes are also implicated in diverse physiological and developmental processes in the uninfected plant. The GH17 family member BGLUII is present in the apoplast of uninfected and infected barley roots and highly induced upon challenge with the pathogen *B. sorokiniana*. The activity of BGLUII on the fungal EPS matrix indicates that its substrate is available/exposed in this layer, the first physical site of contact with the plant defense components. The released β-GD is not immunoactive in barley and Arabidopsis and has some specific properties, which include resilience to further digestion by GH17 family members as well as ROS scavenging abilities. These properties seem to depend on the presence of β-1,6-linked glucosyl substituents. In fact, the structurally related laminariheptaose, which has the same structure as the backbone of β-GD, is highly sensitive to digestion by *Hv*BGLUII and other GH17 family members, is immunoactive in barley, is not significantly degraded/modified in the Fenton reaction and does not display ROS scavenging capabilities (Figure 4D; Supplemental Figures S13 and S14). The removal of the side-branches from the β-GD by a microbial-derived β-1,6-glucanase makes it sensitive to further digestion by GH17 family members, confirming the protective effect displayed by the side-branches. In planta, the β-GD most likely corresponds to a host glucanase-resistant structure because plants are not known to produce β-1,6-glucanases (Fliegmann et al., 2004; Nars et al., 2013).

Additionally, the branching frequency might also be crucial for the immunomodulating activities of β-glucans. In animal systems, glucans are sensed by the well-characterized receptor Dectin-1. The minimal glucan subunit structure for Dectin-1 activation is a β-1,3-glucan oligosaccharide containing a backbone with at least seven glucose subunits and a single β-1,6-linked side-chain branch at the nonreducing end (Adams et al., 2008). We recently have shown that in plants β-1,3-glucan oligomers are perceived in a host species and length-dependent manner. While the monocots barley and *Brachypodium distachyon* can recognize longer (laminarin) and shorter (laminarihexaose) β‐1,3‐glucans with responses of varying intensity, duration and timing, the dicot *Nicotiana benthamiana* activates immunity in response to long β‐1,3‐glucans, whereas Arabidopsis and *Capsella rubella* perceive short β‐1,3‐glucans. The hydrolysis of the β‐1,6 side‐branches of laminarin did not affect recognition, demonstrating that not the glycosidic decoration but rather the degree of polymerization plays a pivotal role in the recognition of long‐chain β‐glucans in plants. Our data do not unambiguously demonstrate the glucosyl position of the substituents on the heptasaccharide backbone of the β-GD, but a possible explanation to the observed differences between the immune properties of laminarin (1:10 branching) and the β-GD (1:2.3 branching) might be that in barley roots β-glucans with a higher degree of branching could stereochemically interfere with each other, leading to less binding by specific receptors. It could well be that the β-GD has different immune properties depending on the plant species and this requires further investigation. However, here we show that upon incubation of β-GD with two unrelated plant species, Arabidopsis and barley, no accumulation of ROS was detected. It should also be mentioned that the antioxidant value of β-GD seems to be strictly “single use” and the β-GD pool may be easily depleted in planta. Nevertheless, a temporary dampening of the oxidative burst may be all that is required to support colonization and/or hamper ROS signaling. The immunomodulatory properties of the β-GD resemble those of fungal effector proteins, suggesting that carbohydrate-class effectors also play an important role in fungal accommodation.

In conclusion, our data demonstrate that the fungal EPS matrix is a source of soluble β-glucans that leads to ROS scavenging properties important for host colonization and represents a distinct outer fungal layer with well-defined protein and carbohydrate signatures. Β-glucans can form complex higher order structures depending on the conformation of sugar residues, molecular weight, inter- and intra-chain hydrogen bonding, and environmental conditions—all effecting their properties. This is different to the immune perception of the MAMP chitin, which is solely based on the length of the released oligomers by, for instance, the activity of chitinases. In plants, β-glucans are considered “orphan MAMPs” as their direct immune receptors have so far not been unambiguously identified. One of the remaining challenges is to fully elucidate the β-glucans’ functions and receptors. Information on the structure, solubility, molecular weight, side-chain branching frequency, and conformation is of paramount importance and should be provided in studies dealing with fungal β-glucans and their effects on the plant immune system.

## Materials and methods

Biological assays were performed with barley (*H. vulgare* L. cv Golden Promise) and *A. thaliana* Col-0 or aequorin-expressing Col-0 lines (Choi et al., 2014). Barley seeds were surface sterilized with 6% bleach for 1 h, followed by five washing steps with 30 mL of sterile Milli-Q water (each 30 min, two quick rinses for a couple of seconds between longer incubation steps). Sterilized seeds were germinated for 3 days on wet filter paper at room temperature in the dark under sterile conditions. Seedlings were transferred to sterile jars containing solid 1/10 plant nutrition medium (PNM), 0.4% gelrite (Duchefa, Haarlem, the Netherlands) Lahrmann et al., 2013) and cultivated in a growth chamber with a day/night cycle of 16 h/8 h (light intensity, 108 μmol  m^−2^ s^−1^) and temperatures of 22°C/18°C. Seedlings were grown for 4 more days before being used for immunity assays.

Arabidopsis seeds were surface sterilized (10 min 70% ethanol, 7 min 100% ethanol) and sown on half-strength Murashige and Skoog (MS) medium (pH 5.7) supplemented with 0.5% sucrose and 0.4% Gelrite (Duchefa, Haarlem, the Netherlands). Plates were transferred to climate chambers with 8-h/16-h light/dark regime (light intensity of 110 μmol m^−2^ s^−1^) at 22°C/18°C. Seven‐day‐old seedlings were transferred into petri dishes filled with 30-mL fresh half-strength MS liquid medium and grown for 7 additional days under the same conditions.

### Commercial enzymes

The commercial β-glucanases used in this study are the *Trichoderma harzianum* lysing enzymes (TLE, Sigma L1412), the *Helix pomatia* β-1,3-glucanase (Sigma 67138) and the *H. vulgare* β-1,3-endoglucanase (GH17 family *Hv*BGLUII, E-LAMHV in 50% (v/v) glycerol, Megazyme). TLE or *H. pomatia* β-1,3-glucanase were prepared in a stock concentration of 1.25 mg·mL^−1^ in respective buffers: TLE (2-mM sodium acetate, pH 5.0), *H. pomatia* β-1,3-glucanase (2-mM MES, pH 5.0). The GH17A family β-1,3-endoglucanase from *Formosa agariphila* (*Fa*GH17a) and the GH30 family β-1,6-exoglucanase from *Formosa* sp. nov strain Hel1_33_131 (*Fb*GH30) were obtained from Prof. Dr Jan-Hendrik Hehemann (Center for Marine Environmental Sciences, University of Bremen, Germany) at a concentration of 5 µg·mL^−1^ in water (Becker et al., 2017). For testing residual glycogen, α-amylase (E-BAASS, Megazyme) was prepared in a stock concentration of 1.25 mg·mL^−1^ using 50% (v/v) glycerol.

### FITC488 labeling and confocal microscopy

FITC488 labeling of *Si*WSC3 and *Si*FGB1 and confocal laser scanning microscopy was done as described previously (Wawra et al., 2019) using the KPL SureLink Fluorescein-X (FAM-X) labeling kit (#82-00-02) by Sera Care with the following modification for *Si*WSC3 labeling: 20-min incubation time at 20°C with half of the recommended concentration. The reaction was stopped by adding 1-M TRIS (pH 7.5) to a final concentration of 50 mM and then dialyzed overnight against 3 L of Milli-Q water. Modifications to the standard labeling protocol were necessary because over labeling reduced the ability of the *Si*WSC3 to bind to its substrate (Wawra et al., 2019).

### Microbial strains and culture conditions for EPS matrix production in *S. indica* and *B. sorokiniana*

The EPS matrix was isolated from the *S. indica* strain expressing GFP in the dikaryotic wild-type background DSM11827 (Wawra et al., 2016) and from the *B. sorokiniana* wild-type strain ND90r. *Serendipita* *indica* spores were isolated from 3-week-old cultures grown on solid complex medium (*Si*_CM) using 0.002% (v/v) Tween water (Zuccaro et al., 2011). For the preculture, 2 mL of *S. indica* spores at a concentration of 500,000 mL^−1^ were inoculated in 100 mL of TSB medium containing 1% (w/v) sucrose and shaking at 120 rpm at 28°C. After 48 h, the pre-cultures were transferred to 400 mL of TSB containing 1% sucrose and shaken at 120 rpm at 28°C for 72 h. *Bipolaris* *sorokiniana* spores were isolated from 10-day-old cultures grown on solid complete medium (*Bs*_CM), using 0.002% (v/v) Tween water (Sarkar et al., 2019). The spores were inoculated at a final concentration of 250 spores·mL^−1^ in 250 mL of YPD medium and these samples were shaken at 28°C for 36 h.

### Isolation of EPS matrix from *S. indica* and *B. sorokiniana* culture supernatant

Culture supernatants from axenically grown *S. indica* or *B. sorokiniana* were collected by filtering the mycelia using Miracloth (Merck Millipore). The EPS matrix was isolated from the culture media using cryogelation. Briefly, the culture media were frozen overnight at −20°C and slowly thawed at room temperature for 16 h. The precipitated EPS matrix (Supplemental Figure S1) present in the culture medium was isolated using a pipette controller and washed four times with 30 mL of Milli-Q water and either used for proteome analyses (see section “Proteome analysis of *S.indica* EPS matrix, CW, and culture filtrate”) or washed one more time with 30 mL of 8.3-mM EDTA (pH 8.0) to remove metal ions potentially present in the EPS. The proteins present in the EPS matrix were removed by treatment with 30 mL of protein denaturation solution (containing 8-M urea, 2-M thiourea, 4% [w/v] sodium dodecyl sulfate [SDS], 100-mM Tris–HCl, pH 7.5) and boiling at 95°C for 15 min. The SDS present in the EPS matrix was removed by boiling the material with 30 mL of Milli-Q water at 95°C for 10 min and centrifuging at 10,000*g* for 10 min at room temperature. The latter step was repeated approximately 15 times until no further foaming occurred. The resulting protein-free EPS matrix was lyophilized and used for glycosyl linkage, TLC or MALDI-TOF analyses.

### Proteome analysis of *S. indica* EPS matrix, CW, and culture filtrate

The proteins were isolated from the EPS matrix, CW, and the culture filtrate obtained from axenic cultures of *S. indica* strain expressing GFP grown in different media (CM, YPD, and TSB).

#### Protein isolation from the CW

Mycelium collected from the *S. indica* GFP strain was ground in liquid N_2_ and resuspended in PBS buffer containing 1-mM PMSF and 1% (v/v) NP-40 using an ULTRA-TURRAX (IKA, Staufen, Germany). The resuspended mixture was incubated at 4°C in a rotating wheel for 30 min. The pellet obtained after centrifugation at 8,000*g* for 15 min at 4°C was resuspended in PBS buffer containing 1-mM PMSF and 0.1% (v/v) IGEPAL using an ULTRA-TURRAX. The pellet obtained after centrifugation at 8,000*g* for 15 min at 4°C was washed three times with Milli-Q water. Finally, the pellet was resuspended in Laemmli buffer containing 8-M urea, 2-M thiourea, and β-mercaptoethanol and boiled at 95°C for 10 min.

#### Protein isolation from the EPS matrix

The EPS matrix obtained from the culture media by cryogelation was washed four times with Milli-Q water and was directly boiled in Laemmli buffer containing 8-M urea, 2-M thiourea, and β-mercaptoethanol at 95°C for 10 min.

#### Protein isolation from the culture filtrate

The culture supernatant left over from the EPS matrix isolation step was first filtered using a Whatman filter paper and then using a 0.22-µM syringe filter. Approximately 30 mL of EPS matrix depleted culture supernatant was treated with 5 mL of 95% (v/v) trichloroacetic acid and incubated overnight at 4°C. The precipitated proteins were collected by centrifugation (10,000*g*) for 1 h at 4°C. The isolated proteins were washed at least three times with 100% (v/v) acetone. The dried protein pellet was resuspended in Laemmli buffer containing 8-M urea, 2-M thiourea, and β-mercaptoethanol and boiled at 95°C for 10 min.

### Liquid chromatography coupled mass spectrometric protein identification and quantification

The proteins isolated from the EPS matrix, CW and culture filtrate were separated using a 10% (v/v) SDS–polyacrylamide gel electrophoresis gel for 15 min and subsequently stained with Coomassie Brilliant Blue. Protein-containing bands from Coomassie-stained gels were prepared for mass spectrometric analysis as described elsewhere (Poschmann et al., 2014). Briefly, bands were destained and the proteins were reduced with dithiothreitol and alkylated with iodoacetamide and subjected to tryptic digestion. The resulting peptides were extracted and reconstituted in 0.1% (v/v) trifluoroacetic acid in water. Peptides were separated on an Ultimate 3000 Rapid Separation Liquid Chromatography system (Thermo Fisher Scientific) on a 25-cm length C18 column using a 1-h gradient and subsequently analyzed by a QExactive Plus mass spectrometer (Thermo Fisher Scientific) as described with minor modifications (Poschmann et al., 2014). First, survey scans were carried out at a resolution of 140,000 and up to 22- and 3-fold charged precursors selected by the quadrupole (4 *m/z* isolation window), fragmented by higher energy collisional dissociation and fragment spectra recorded at a resolution of 17,500. Mass spectrometric data were further processed by MaxQuant 1.6.12.0 (Max-Planck Institute for Biochemistry, Planegg, Germany) with standard parameters if not otherwise stated. Label-free quantification, “match between runs” and iBAQ quantification were enabled. Searches were carried out based on *S. indica* reference protein entries (UP000007148), downloaded on 15 May 2020 from the UniProt Knowledge Base. Carbamidomethylation at cysteines was considered as fixed and methionine oxidation and proteins N-terminal acetylation as variable modifications. Peptides and proteins were accepted at a false discovery rate of 1% and only proteins further considered were identified with at least two different peptides. The identified proteins were grouped into families using the Pfam database (Mistry et al., 2021). The mass spectrometry proteomics data have been deposited to the ProteomeXchange Consortium via the PRIDE partner repository with the dataset identifier PXD025640.

### Proteome analysis of *S. indica* EPS matrix, CW, and culture filtrate

Further calculations were done on label-free quantification (LFQ) intensity values. Only proteins were considered showing at least two valid intensity values at least in one group. LFQ intensity values were log2 transformed and missing values imputed with values drawn from a downshifted normal distribution (width 0.3, downshift 1.8) before statistical and enrichment analysis. Statistical analysis was done for selected protein groups using the significance analysis of microarrays method (Tusher et al., 2001; 5% false discovery rate, S0 = 0.1) and Student’s *t* test. To identify proteins containing certain domains showing a higher abundance in the EPS matrix, we performed an annotation enrichment analysis (Cox and Mann, 2012) based on the differences of mean values of log2 transformed LFQ intensities.

The percentage relative abundance of signal peptide containing proteins detected in the three components (EPS matrix, CW, and culture filtrate) was calculated using LFQ intensities.

### Large scale digestion and enrichment of the β-GD released from the EPS matrix of *S. indica* and *B. sorokiniana*

Five milligram of freeze-dried EPS matrix obtained from *S. indica* or *B. sorokiniana* were ground with two stainless steel beads (5 mm) using a TissueLyser (Qiagen, Hilden, Germany) at 30 Hz for 1 min at room temperature and soaked in 1 mL of 100-mM sodium acetate buffer (pH 5.0) at 70°C overnight. The soaked material was incubated with 20 µL (50 U) of *Hv*BGLUII in a total reaction volume of 1 mL using 100-mM sodium acetate buffer (pH 5.0). The digestion was performed at 40°C with shaking at 500 rpm for 48 h. The supernatant containing the β-GD was collected by centrifugation at 10,000*g* for 5 min at room temperature. The digested pellet was additionally suspended in 1 mL of water and heated at 80°C for 10 min to solubilize additional β-GD. The resulting supernatant was combined with the initial digested material and boiled at 95°C for 15 min. The precipitated proteins were removed by centrifugation at 10,000*g* for 20 min at room temperature and the debris were removed using a 0.45-µM syringe filter. The clear supernatant fraction was freeze-dried. The freeze-dried material was dialyzed against 3 L of Milli-Q water at 4°C using a 1-kDa cutoff dialysis tubing (Repligen Spectra/Por 6 Pre-Wetted Regenerated Cellulose, cat. no. 888-11461). The dialyzate was lyophilized before further use.

### Preparation of alcohol insoluble residue and protein-free CW from *S. indica* and *B. sorokiniana*

The mycelium collected from axenic cultures of *S. indica* or *B. sorokiniana* was washed twice with Milli-Q water and freeze-dried overnight. The freeze-dried material was powdered in liquid N_2_ using a pestle and mortar and stored at −20°C until use. Twenty milligrams of the material was resuspended in 1 mL of 70% (v/v) aqueous ethanol with a stainless steel bead (5 mm) using a TissueLyser at 30 Hz for 1 min. After centrifugation at 10,000*g* for 20 min at room temperature, the pellet was washed with chloroform/methanol (1:1), then acetone and subsequently air-dried. Protein-free fungal wall material was prepared as mentioned previously (Wawra et al., 2016).

### Glycosyl linkage analysis of EPS matrices and CW preparations

EPS matrix (2 mg) or CW preparation of *S. indica* or *B. sorokiniana* were ground with a stainless steel bead (5 mm) using a TissueLyser mill at 30 Hz for 1 min. The powdered material was subjected to glycosyl linkage analysis as described (Liu et al., 2015). Briefly, a methylation reaction was performed using NaOH/DMSO. The methylated compounds were hydrolyzed in 1 M trifluoroacetic acid, reduced using sodium borodeuteride (ACROS Organics, cat.no. 194950050) and per-o-acetylated. The resulting partially methylated alditol acetates were analyzed using an Agilent 5977A GC/MSD System equipped with a SP-2380 Fused Silica Capillary Column (Supelco). The glycosidic linkages were assigned based on retention time and mass spectrum fragmentation patterns compared to the CCRC spectral database (https://www.ccrc.uga.edu/specdb/ms/pmaa/pframe.html).

### Digestion of EPS matrix and CW and TLC analysis

Freeze-dried EPS matrix (1 mg) or CW preparation (1 mg) from *S. indica* or *B. sorokiniana* were suspended in 400 µL of 2-mM sodium acetate (pH 5.0; for TLE), 2-mM MES (pH 5.0; for *H. pomatia* β-1,3-glucanase), or 100-mM sodium acetate (pH 5.0; for *Hv*BGLUII) at 70°C overnight. The excess buffer was removed and the suspended material was treated with 5 µL of the glucanase enzymes (0.125 mg·mL^−1^ or 12.5 U for *Hv*BGLUII) in the respective buffers, containing 1 µL of BSA (100 mg·mL^−1^) in a total reaction volume of 50 µL and incubated at 40°C by shaking at 500 rpm for 16 h. The digestion reaction was stopped by incubating the samples at 95°C for 10 min. An aliquot was subjected to TLC using a silica gel 60 F254 aluminum TLC plate (Merck Millipore, cat. no. 105554), with a running buffer containing ethyl acetate/acetic acid/methanol/formic acid/water in a ratio of 80:40:10:10:10 (v/v). D-glucose, laminaribiose β-1-3-(Glc)_2_, laminaritriose β-1-3-(Glc)_3_, gentiobiose β-1-6-(Glc)_2_, and laminaripentaose β-1-3-(Glc)_5_ with a concentration of 1.5 mg·mL^−1^ were used as standards. The glucan fragments were visualized by spraying the TLC plate with glucan developer solution (containing 45-mg N-naphthol, 4.8-mL H_2_SO_4_, 37.2-mL ethanol, and 3-mL water) and heating the TLC plate to 100°C until the glucan bands were visible (∼4–5 min).

### MALDI-TOF analysis

#### Analysis of the β-GD from S. indica

Freeze-dried β-GD was solubilized in water at 70°C for 10 min.

#### Structural characterization of the β-GD from S. indica and B. sorokiniana

Ten microliters of β-GD (2 mg·mL^−1^) was treated with 2 µL of *Fa*GH17a or 2-µL *Fb*GH30 or 1 µL of *Fa*GH17a + 1 µL *Fb*GH30 in 50 µL of Milli-Q water. The digestion reaction was carried out at 40°C for 16 h and the reaction was stopped by incubation at 95°C for 5 min.

#### Analysis of oligosaccharides released from the EPS matrix and CW of S. indica

Freeze-dried EPS matrix (1 mg) or CW preparation (1 mg) isolated from *S. indica* were suspended in 400 µL of a 2-mM sodium acetate buffer, pH 5.0 (TLE), 2-mM MES buffer, pH 5.0 (*H. pomatia* β-1,3-glucanase), or 25-mM sodium acetate buffer, pH 5.0 (*Hv*BGLUII) and incubated at 70°C overnight. The suspended material was treated with 2.5 µL of TLE, 2.5 µL of *H. pomatia* β-1,3-glucanase, or 1 µL of *Hv*BGLUII in the respective buffers, as described before, in a total reaction volume of 50 µL. The digestion was performed at 40°C with shaking at 500 rpm for 16 h. The digestion reaction was stopped at 95°C for 10 min and centrifuged at 11,000*g* for 5 min at room temperature.

#### Analysis of oligosaccharides released from the EPS matrix and CW of B. sorokiniana

Freeze-dried EPS matrix (1 mg) or CW preparation (1 mg) isolated from *B. sorokiniana* were solubilized in 400 µL of 2-mM sodium acetate, pH 5.0 (TLE) or 25-mM sodium acetate, pH 5.0 (*Hv*BGLUII). The solubilized material was treated with 1 µL of TLE or 1 µL of *Hv*BGLUII in a total reaction volume of 50 µL. The digestion was performed at 40°C with shaking at 500 rpm for 16 h. The digestion reaction was stopped at 95°C for 10 min.

#### Mass spectrometrical analysis

The oligosaccharides present in the prepared samples were analyzed by Oligosaccharide Mass Profiling as described (Günl et al., 2011). Briefly, the samples were spotted onto a dried spot of dihydroxy benzoic acid matrix (10 mg·mL^−1^) and analyzed by MALDI-TOF mass spectrometry (Bruker rapifleX instrument). The machine was set to linear, positive reflectron mode with an accelerating voltage of 20,000 V. The spectra from the samples were analyzed using flexanalysis software 4.0 (Bruker Daltonics).

### Reduction and purification of *S. indica* β-GD

Enriched β-GD (40 mg) was reduced with sodium borodeuteride (20 mg·mL^−1^; ACROS Organics, cat.no. 194950050) in 1-M ammonium hydroxide for 90 min at room temperature. The reaction was neutralized by addition of glacial acetic acid and 9:1 (v:v) methanol:acetic acid. The solvents were evaporated under N_2_ gas. The dried material was washed once with 9:1 (v:v) methanol:acetic acid and three times with methanol. In each washing step, the methanol:acetic acid or methanol were evaporated under N_2_ gas. The dried material was dissolved in 6% (v/v) aqueous methanol, vortexed, and centrifuged at 4,000*g* for 15 min at room temperature, to remove any occurring debris. The supernatant (50 µL) was subjected to reverse-phase chromatography using a Vydac 238 TP C18 column (Vydac, Hesperia, CA, USA) eluting with a linear gradient from 6% (v/v) to 12% (v/v) methanol in 10 min, followed by 12% (v/v) to 50% (v/v) methanol in 10 min and equilibrated back to 6% (v/v) methanol in 10 min with a flow rate of 0.5 mL·min^−1^. The eluting compounds were detected by an evaporative light scattering detector (ERC GmbH, Munich, Germany) at 38°C and simultaneously collected for MALDI-TOF analyses. Collected fractions containing the β-GD were pooled and freeze-dried.

### ^1^H NMR analysis

Reduced β-GD (1 mg) was dissolved in D_2_O (100% atom D, ACROS Organics, 320700075) at 80°C for 10 min and subsequently freeze-dried overnight. The freeze-dried material was dissolved in 300 µL of 6:1 (v/v) of methyl sulphoxide D6 (99.9% atom D + 1% tetramethylsilane, ACROS Organics):D_2_O (100% atom D, ACROS Organics, 320700075) at 80°C for 10 min. The ^1^H NMR spectrum of the reduced β-GD was measured using a 600 MHz Bruker NMR spectrometer at 80°C (Kim et al., 2000). The chemical shift signal was referred to the internal standard tetramethylsilane at 0 ppm and the ^1^H NMR spectrum was processed using Bruker’s Topspin software.

### Purification of native β-GD for the ROS burst assay

Enriched β-GD (40 mg) was dissolved in 6% (v/v) aqueous methanol, vortexed, and centrifuged to remove any debris. The β-GD was purified as described above but without reduction and used for the ROS burst assays if not otherwise stated in the legend.

### Preparation of crude fungal CW substrates for immunity assays

For crude extraction of soluble fragments from the fungal CW and EPS matrix, 5 mg of the respective fungal substrate was transferred to a 2-mL Eppendorf tube with three stainless steel beads (5 mm), snap-frozen in liquid nitrogen and ground with a TissueLyser (1 min, 30 Hz). The ground substrate was resuspended in 2 mL of Milli-Q water, incubated at 70°C for 16 h (700 rpm), and then boiled at 95°C for 10 min. The volume was filled up to 5 mL for a final concentration of 1 mg·mL^−1^. The suspension was centrifuged at 13,000*g* for 10 min at room temperature and the supernatant was further used in plant immunity assays.

### Plant immunity assays

#### Preparation of immunity assays

For immunity assays using barley, roots were separated from 7-day-old seedlings (cut 2 cm below seed), root tips were removed (first 1 cm), and residual roots were cut into 5-mm pieces. Each assay was performed with randomized root pieces from 16 barley seedlings. Four root pieces were transferred into each well of a 96-well plate microtiter plate containing Milli-Q water. For immunity assays with Arabidopsis (wild-type or aequorin-expressing lines), intact 14-day-old seedlings were transferred into a 96-well plate (one seedling/well) filled with Milli-Q water.

#### Elicitors

Chitohexaose was purchased from Megazyme (Bray, Ireland), flg22 from GenScript (Piscataway, NJ, USA), and laminarin from Sigma‐Aldrich (Taufkirchen, Germany). All commercial substrates were dissolved in Milli-Q water without additional treatment. Substrates derived from fungal CW and EPS were prepared as described earlier. The elicitor solutions had the following pH ± sd (*n* = 3): Milli-Q water = 7.20 ± 0.125; EPS matrix prep1 = 7.30 ± 0.322; EPS matrix prep2 = 7.37 ± 0.167; β-GD = 6.96 ± 0.212; Chit6 = 7.32 ± 0.106.

#### Cytosolic calcium influx measurements

The solution in the 96-well plates containing the plant material (Arabidopsis seedlings expressing aequorin, see above) was exchanged for 100 µL of a 10-µM coelenterazine solution (Roth, Karlsruhe, Germany). After overnight incubation in the dark, the plates were transferred to a TECAN SPARK 10M multi-well plate reader. After baseline measurement (5 min), 50 µL of a three-fold concentrated elicitor solution was manually added to the wells and luminescence was measured for 30 min. Residual aequorin was discharged with 100 μL of 2.5-m CaCl_2_ (in 25% [v/v] ethanol) and luminescence was detected for 1 min. All measurements were performed with an integration time of 300 ms. Baseline luminescence and elicitor-triggered luminescence were normalized according to the maximal integrated discharge value obtained from individual wells.

#### ROS assay

The ROS assay is based on previously published protocols (Felix et al., 1999; Wanke et al., 2020; Ngou et al., 2021). In detail, 96-well plate with plant material (see above) were incubated overnight at room temperature on the bench to remove ROS and other contaminants originating from the mechanical damage of the tissue during preparation. The next day, water was replaced by 100 µL of fresh Milli-Q water containing 20 µg·mL^−1^ horseradish peroxidase (Sigma‐Aldrich, Taufkirchen, Germany) and 20-µM L‐012 (Wako Chemicals, Neuss, Germany). Following a short incubation time (∼15 min), 100 µL of double-concentrated elicitor solutions were added to the wells. Measurements of elicitor-triggered apoplastic ROS production were started immediately and taken continuously with an integration time of 450 ms using a TECAN SPARK 10 M multi-well plate reader. Horseradish peroxidase catalyzes the oxidation of the luminol derivate L-012 by the plant-produced ROS species upon elicitation with MAMPs. This leads to a chemiluminescent signal that is detected by the plate reader.

#### Gene expression analysis

After the ROS burst assay (1 h after elicitor addition), plant material from three to four wells was pooled, dried on tissue paper and snap-frozen in liquid nitrogen for further analysis of gene expression changes. Total RNA was extracted using TRIzol reagent (Invitrogen, Karlsruhe, Germany) and contaminating gDNA was digested during a DNaseI treatment (Thermo Fisher Scientific, Schwerte, Germany) according to manufacturers’ instructions. Synthesis of cDNA was carried out using the First Strand cDNA Synthesis Kit (Thermo Fisher Scientific, Schwerte, Germany) without changes to the manufacturer’s protocol. Target gene expression was analyzed by reverse transcription-quantitative polymerase chain reaction (RT-qPCR) as described previously (Wawra et al., 2019). Relative expression of elicitor-responsive *HvWRKY2* gene (Shen et al., 2007) compared to *H. vulgare* ubiquitin gene (Sarkar et al., 2019) was determined using the following primer pairs: *HvUBI*_F (5′-ACCCTCGCCGACTACAACAT-3′) with *HvUBI*_R (5′-CAGTAGTGGCGGTCGAAGT-3′) and *HvWRKY2*_F (5′-AACAACCACCACCAGTCGTT-3′) with *Hv*WRKY2_R (5′-TCACCTTCTGCCCGTACTTC-3′). Gene expression levels in elicitor-treated samples were normalized to the expression levels in mock-treated samples.

### *In vitro* DAB oxidation assay

DAB assay was performed as previously described (Nostadt et al., 2020). Sugar substrates (concentrations as indicated) were mixed with 0.05-µM horseradish peroxidase (Sigma‐Aldrich, Taufkirchen, Germany) and 1-mM H_2_O_2_ (Sigma‐Aldrich, Taufkirchen, Germany) in reaction buffer (50-mM sodium acetate, 150-mM NaCl, pH 5) and incubated for 10 min at room temperature in a 96-well plate. Then, an equal volume of a 200-µM DAB solution was added to the wells. After 16 h of incubation in the dark, plates were scanned on a flatbed scanner (transmissive light mode).

### Fenton reaction-based oxidation of sugars

The assay was performed as previously described (Matros et al., 2015). Carbohydrate samples (300 µM) were mixed with Fenton reagents (1-mM H_2_O_2_ [Sigma], 100-µM FeSO_4_ [Sigma]) and incubated overnight at 30°C. Control samples with an additional 100-µM EDTA (Sigma) or without Fenton reagents were treated in the same way. Samples were centrifuged at 13,000*g* for 10 min at room temperature and the supernatant was further analyzed by MALDI-TOF.

### Colonization assay with β-GD

Roots of 4-day-old barley seedlings were inoculated with 3 mL of *S. indica* spores at a concentration of 500,000 mL^−1^ and grown at a day/night cycle of 16 h/8 h at 22°C/18°C, 60% humidity, and 108 µmol m^−2^ s^−1^ light intensity. At 1 and 2 dpi, 1 mL of sterile water as a control or 100- or 300-µM β-GD were added to the jars which contained four seedlings each. The seedlings of each jar were pooled and harvested at 3 dpi. The roots were washed in ice water to remove extraradical hyphae, cut as previously described (Nizam et al., 2019), frozen in liquid nitrogen and used for RNA extraction. RNA extraction for fungal colonization, cDNA generation, and RT-qPCR was performed as previously described (Sarkar et al., 2019). For quantification of endophytic fungal colonization by RT-qPCR, the following primers were used: 5′-GCAAGTTCTCCGAGCTCATC-3′ and 5′-CCAAGTGGTGGGTACTCGTT-3′ for *S. indica* translation-elongation factor (*SiTEF*) and 5′-ACCCTCGCCGACTACAACAT-3′ and 5′CAGTAGTGGCGGTCGAAGTG3′ for barley ubiquitin (*HvUbi*).

### Expression analysis of selected genes with WSC domains

Gene expression of *S. indica* WSC domain-containing proteins during root colonization of barley and Arabidopsis was monitored via RT-qPCR as previously described (Wawra et al., 2019) at 3, 7, and 14 dpi. For each biological replicate (*n* = 3), roots from several individual plants were pooled (Arabidopsis: 60 plants; barley: 4 plants).

### Statistical analyses

A summary of statistical analyses is given in Supplemental Data Set S10.

### Accession numbers

***Hordeum vulgare* BGLU2**: P15737 (UniProt), HORVU.MOREX.r3.3HG0319100.1 (MorexV3_pseudomolecules_assembly, EnsemblePlants).

## Supplemental data

The following materials are available in the online version of this article.

**Supplemental Figure S1.** EPS matrix isolated from *S. indica* grown in CM medium using a cryogelation approach.

**Supplemental Figure S2.** Proteome analysis of *S. indica* EPS matrix, CW, and culture filtrate.

**Supplemental Figure S3.** Expression analysis of selected genes with WSC domains in *S. indica* during plant colonization at 3, 7, and 14 dpi.

**Supplemental Figure S4.** Glycosyl linkage analysis of *Si* EPS and *Si* CW.

**Supplemental Figure S5.** Analysis of oligosaccharides released from the EPS or protein-free CW of *S. indica* by the action of β-1,3-glucanases.

**Supplemental Figure S6.** Glycosyl linkage and MALDI-TOF analysis of the glucan fraction (β-GD) released from the EPS matrix of *S. indica.*

**Supplemental Figure S7.** Purification of the β-GD fragment for ^1^H NMR analysis.

**Supplemental Figure S8.** Mechanically released fragments from *S. indica* EPS matrix and CW layer do not exhibit ROS scavenging activity.

**Supplemental Figure S9.** Chitohexaose-triggered ROS accumulation is decreased by *S. indica* β-GD treatment in a concentration-dependent manner.

**Supplemental Figure S10.** Purification of native β-GD and immunogenic characterization.

**Supplemental Figure S11.** Detoxification of apoplastic ROS by *S. indica* β-GD is independent of elicitor treatment and plant species.

**Supplemental Figure S12.** *Serendipita indica* β-GD treatment does not trigger cytosolic calcium influx in *A. thaliana* seedlings.

**Supplemental Figure S13.** Digestion of laminariheptaose with *Hv*BGLUII enhances ROS production in barley roots.

**Supplemental Figure S14.** Glycan controls are not degraded by hydrogen peroxide during Fenton reaction.

**Supplemental Figure S15.** Glycosyl linkage analysis of *B. sorokiniana* EPS matrix and CW.

**Supplemental Figure S16.** Analysis of oligosaccharides released from the EPS of *B. sorokiniana* by the action of *Trichoderma harzianum* lysing enzymes.

**Supplemental Figure S17.** The β-GD released from the EPS matrix of *B. sorokiniana* consists of a seven unit β-1,3-linked glucan backbone substituted with three β-1,6-glucosyl residues.

**Supplemental Figure S18.** Mechanically released fragments from *B. sorokiniana* EPS matrix or CW do not scavenge ROS.

**Supplemental Data Set S1**. Total proteins detected in the proteome analysis of the EPS matrix, CW, and culture filtrate isolated from *S. indica* grown in CM, TSB, or YPD liquid media.

**Supplemental Data Set S2.** Relative abundance of signal peptide (SP) containing proteins detected in the EPS matrix, CW, and culture filtrate isolated from the *S. indica* under three culture mediums: CM, TSB, and YPD. LFQ intensities of the proteins were used to calculate their relative abundance.

**Supplemental Data Set S3**. Statistics, enrichment score, Benjamini–Hochberg corrected *P*-value.

**Supplemental Data Set S4**. Glycosyl sugar residues detected in the total ion chromatogram of EPS matrix isolated from *S. indica*, *n* = 4 independent biological replicates.

**Supplemental Data Set S5**. Glycosyl sugar residues detected in the total ion chromatogram of alcohol insoluble residue (cell wall) isolated from *S. indica*, *n* = 4 independent biological replicates.

**Supplemental Data Set S6.** List of Barley CAZymes detected in the root apoplastic fluids (AFs) of barley after mock, *S. indica* or *B. sorokiniana* treatments.

**Supplemental Data Set S7**. Glycosyl sugar residues detected in the total ion chromatogram of alcohol insoluble residue (cell wall) isolated from *B. sorokiniana*, *n* = 3 independent biological replicates.

**Supplemental Data Set S8**. Glycosyl sugar residues detected in the total ion chromatogram of EPS matrix isolated from *B. sorokiniana*, *n* = 3 independent biological replicates.

**Supplemental Data Set S9**. List of Barley CAZymes differentially regulated during *B. sorokiniana* infection.

**Supplemental Data Set S10.** Summary of statistical analyses.

## Supplementary Material

koac114_Supplementary_Data

## Notes

These authors contributed equally (B.C., A.W. and S.W.).

B.C., A.W., S.W., and A.Z. conceived the study. S.W. established the protocols for the CW and EPS matrix extraction and performed the proteomics of EPS, CW, and culture filtrates together with G.P. and K.S. B.C. performed carbohydrate analytics (glycosyl linkage analysis of EPS and CW, MALDI-TOF, purification of β-GD for ^1^H NMR, and ROS burst assay). A.W. performed DAB assays and gene expression analysis. A.W. and B.C. performed β-GD oxidation assays. A.W., S.W., P.S., M.N., M.M., M.T., N.C. performed the ROS burst assays, EPS and CW preparations, and enzymatic digestions. L.M. performed the colonization assays. M.D. contributed to the ^1^H NMR result interpretation. M.P. directed and supervised the carbohydrate analytics. A.Z. supervised the project, designed the experiments, and wrote the paper with contribution of all the authors.

The author responsible for distribution of materials integral to the findings presented in this article in accordance with the policy described in the Instructions for Authors (https://academic.oup.com/plcell) is: Alga Zuccaro (azuccaro@uni-koeln.de).
