# Supplementary material for: Fungi hijack a ubiquitous plant apoplastic endoglucanase to release a ROS scavenging β-glucan decasaccharide to subvert immune responses
Source: Plant Cell. 2022 Apr 20;34(7):2765–84. doi: 10.1093/plcell/koac114 (PMC9252488; doi:10.1093/plcell/koac114)
Supplement: koac114_Supplementary_Data [file koac114_supplementary_data.zip › koac114-suppl_data/tpc.21.00790_Supplemental Data.pdf]

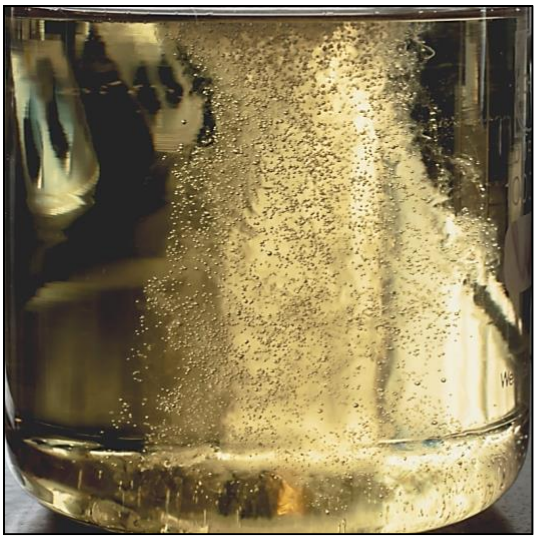

Soluble extracellular polysaccharide (EPS) matrix

Supplemental Figure S1. EPS matrix isolated from *S. indica* grown in CM medium using a cryogelation approach. Supports Figure 2. Air bubbles were embedded in the gel-like EPS matrix after stirring to make the EPS matrix visible.

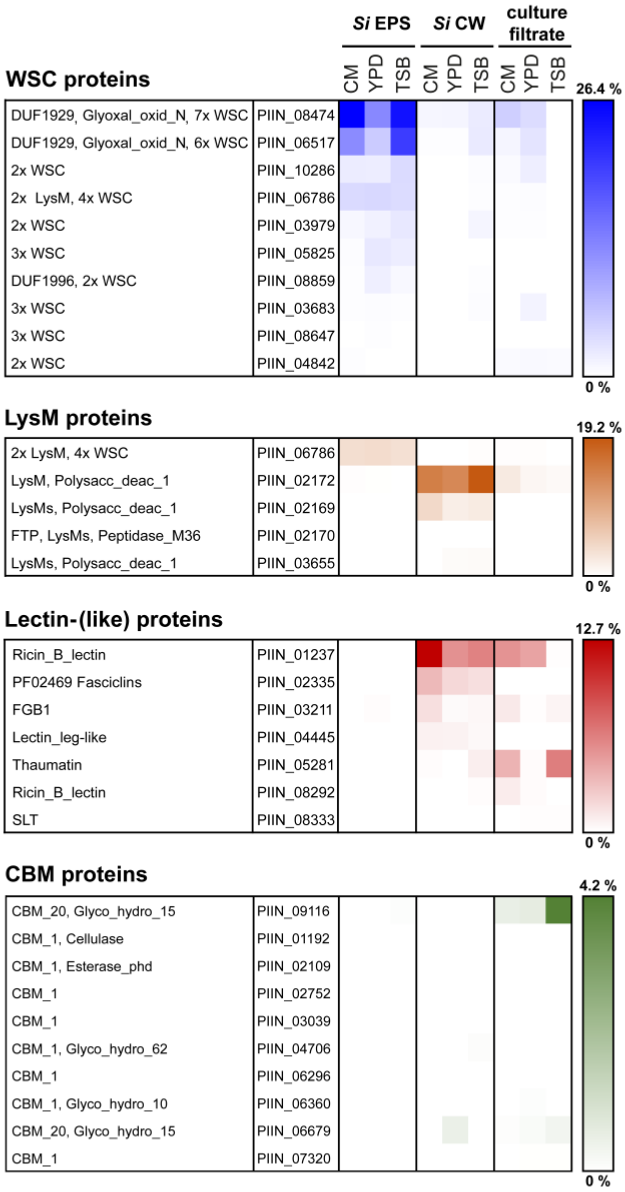

% of proteins with predicted signal peptide

WSC proteins

		Si EPS CM	*Si* EPS YPD	*Si* EPS TSB	*Si* CW CM	*Si* CW YPD	*Si* CW TSB	culture filtrate CM	culture filtrate YPD	culture filtrate TSB
DUF1929, Glyoxal_oxid_N, 7x WSC	PIIN_08474	26.40	12.28	24.67	0.90	1.00	2.04	4.93	3.54	0.05
DUF1929, Glyoxal_oxid_N, 6x WSC	PIIN_06517	11.93	5.31	20.29	0.12	0.10	2.12	0.93	2.76	0.06
2x WSC	PIIN_10286	1.94	1.77	3.65	...	...	0.26	0.47	1.70	0.01
2x LysM, 4x WSC	PIIN_06786	3.74	3.97	3.53	...	...	0.20	0.13	0.21	...
2x WSC	PIIN_03979	0.74	1.36	2.34	...	...	1.00	0.13	0.11	...
3x WSC	PIIN_05825	0.22	2.36	1.83	...	...	0.03	...	...	...
DUF1996, 2x WSC	PIIN_08859	0.15	1.58	0.63	...	...	0.14	...	0.03	...
3x WSC	PIIN_03683	0.14	0.21	0.12	0.05	...	0.26	...	1.24	0.03
3x WSC	PIIN_08647	0.01	0.13	0.05	...	...	0.02	...	...	...
2x WSC	PIIN_04842	0.15	...	...	...	...	...	0.51	0.57	0.46
**Subtotal (%)**		**45.41**	**28.96**	**57.10**	**1.08**	**1.10**	**6.08**	**7.09**	**10.16**	**0.62**

LysM proteins

		Si EPS CM	*Si* EPS YPD	*Si* EPS TSB	*Si* CW CM	*Si* CW YPD	*Si* CW TSB	culture filtrate CM	culture filtrate YPD	culture filtrate TSB
2x LysM, 4x WSC	PIIN_06786	3.74	3.97	3.53	...	...	0.20	0.13	0.21	...
LysM, Polysacc_deac_1	PIIN_02172	0.12	0.09	0.03	14.92	13.71	19.20	2.40	0.94	0.60
LysMs, Polysacc_deac_1	PIIN_02169	...	0.01	...	4.33	1.89	2.28	0.02	...	...
FTP, LysMs, Peptidase_M36	PIIN_02170	...	...	...	...	...	...	0.03	0.03	0.02
LysMs, Polysacc_deac_1	PIIN_03655	0.03	...	...	...	0.43	0.52	...	0.03	0.01
**Subtotal (%)**		**3.90**	**4.06**	**3.56**	**19.25**	**16.02**	**22.21**	**2.58**	**1.20**	**0.64**

Lectin-(like) proteins

		Si EPS CM	*Si* EPS YPD	*Si* EPS TSB	*Si* CW CM	*Si* CW YPD	*Si* CW TSB	culture filtrate CM	culture filtrate YPD	culture filtrate TSB
Ricin_B_lectin	PIIN_01237	0.03	0.05	...	12.71	5.47	6.23	5.34	4.57	0.09
PF02469 Fasciclins	PIIN_02335	...	...	...	3.49	1.92	1.58	...	...	...
FGB1	PIIN_03211	...	0.11	...	1.57	0.24	0.36	1.08	0.05	0.54
Lectin_leg-like	PIIN_04445	...	0.01	...	0.68	0.63	0.34	...	...	...
Thaumatin	PIIN_05281	0.01	...	...	0.12	...	0.85	3.76	0.18	6.47
Ricin_B_lectin	PIIN_08292	...	...	...	...	...	0.12	0.93	0.16	0.03
SLT	PIIN_08333	...	...	...	...	...	...	...	0.05	0.05
**Subtotal (%)**		**0.04**	**0.17**	**0.00**	**18.58**	**8.26**	**9.49**	**11.11**	**5.01**	**7.19**

CBM proteins

		Si EPS CM	*Si* EPS YPD	*Si* EPS TSB	*Si* CW CM	*Si* CW YPD	*Si* CW TSB	culture filtrate CM	culture filtrate YPD	culture filtrate TSB
CBM_20, Glyco_hydro_15	PIIN_09116	...	...	0.06	...	...	...	0.52	0.61	4.20
CBM_1, Cellulase	PIIN_01192	...	...	...	...	...	...	...	0.02	...
CBM_1, Esterase_phd	PIIN_02109	...	...	...	...	...	...	...	0.01	0.02
CBM_1	PIIN_02752	...	...	...	...	...	...	...	0.01	...
CBM_1	PIIN_03039	...	...	...	...	...	...	...	...	0.01
CBM_1, Glyco_hydro_62	PIIN_04706	...	...	...	...	...	0.07	...	0.01	...
CBM_1	PIIN_06296	...	...	...	...	...	...	...	0.02	...
CBM_1, Glyco_hydro_10	PIIN_06360	...	...	...	...	...	...	0.01	0.05	0.01
CBM_20, Glyco_hydro_15	PIIN_06679	...	...	...	...	0.48	...	0.05	0.13	0.31
CBM_1	PIIN_07320	...	...	...	...	...	...	...	0.02	0.02
**Subtotal (%)**		**0.00**	**0.00**	**0.06**	**0.00**	**0.48**	**0.07**	**0.58**	**0.89**	**4.57**

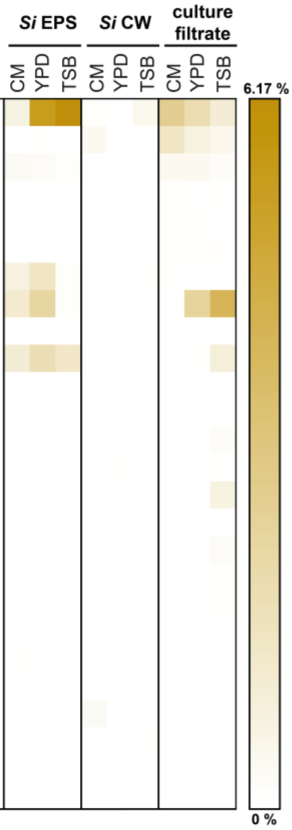

% of proteins with predicted signal peptide

Proteases

		Si EPS			*Si* CW			culture filtrate		
		CM	YPD	TSB	CM	YPD	TSB	CM	YPD	TSB
fn3_5, Peptidase_S8	PIIN_02492	0.68	5.50	6.17	0.16	...	0.41	2.69	1.91	1.05
Peptidase_S10	PIIN_07689	0.04	0.10	...	0.38	...	...	1.47	0.76	0.46
Peptidase_S8	PIIN_04017	0.29	0.24	0.15	...	...	0.03	0.35	0.35	0.17
Peptidase_M28	PIIN_04685	...	...	...	0.01	...	...	0.12	0.02	0.09
FTP, Peptidase_M36	PIIN_11737	...	...	...	...	...	...	0.09	0.05	0.04
Peptidase_M43	PIIN_07555	...	...	...	...	0.01	...	0.06	0.14	0.12
fn3_5, Peptidase_S8	PIIN_05468	0.75	1.45	0.13	...	...	0.10	0.06	0.04	0.01
Peptidase_S41	PIIN_10082	1.18	2.26	0.12	0.04	...	...	0.04	2.41	4.11
FTP, LysMs, Peptidase_M36	PIIN_02170	...	...	...	...	...	...	0.03	0.03	0.02
Peptidase_S28s	PIIN_01377	1.00	1.82	1.33	...	...	0.04	0.03	0.06	0.92
Peptidase_S10	PIIN_06875	...	...	...	...	...	...	0.03	0.05	0.02
Inhibitor_I9, Peptidase_S8	PIIN_07492	0.02	...	...	...	...	...	0.02	0.02	0.03
FTP, Peptidase_M36	PIIN_00424	...	...	...	...	...	...	0.01	0.01	0.17
Peptidase_M28	PIIN_04932	...	...	...	...	0.06	...	0.01	0.01	0.08
FTP, Peptidase_M36	PIIN_09051	0.02	...	0.03	...	...	...	0.01	0.01	0.71
Peptidase_M35	PIIN_06258	...	...	...	0.01	...	...	...	...	0.03
Peptidase_M14, Propep_M14	PIIN_00112	...	...	...	...	0.02	...	...	...	0.23
Peptidase_M28	PIIN_04931	...	...	...	...	...	...	...	...	0.14
Peptidase_M43	PIIN_02952	...	...	...	...	...	...	...	...	0.13
FTP, Peptidase_M36	PIIN_02992	0.01	...	...	...	...	0.01	...	0.02	0.04
FTP, Peptidase_M36	PIIN_05476	0.07	0.02	...	...	...	0.05	...	...	0.02
Inhibitor_I9, Peptidase_S8	PIIN_07598	...	...	...	...	...	...	...	...	0.01
Peptidase_M28	PIIN_01065	0.05	...	...	0.28	...	...	...	...	...
Peptidase_M28	PIIN_00565	...	0.01	...	...	...	0.08	...	...	...
Peptidase_S8	PIIN_04374	...	...	...	...	...	...	...	...	...
Peptidase_S66, Peptidase_S66C	PIIN_00863	0.01	...	...	...	...	...	...	...	...
**Subtotal (%)**		**4.12**	**11.40**	**7.95**	**0.87**	**0.09**	**0.73**	**5.05**	**5.89**	**8.60**

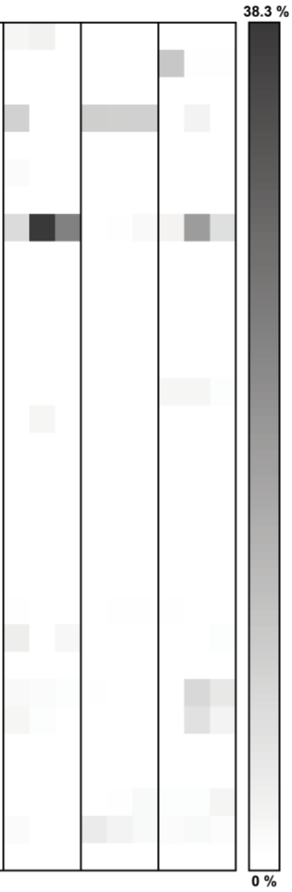

CAZymes

		Si EPS			*Si* CW			culture filtrate		
		CM	YPD	TSB	CM	YPD	TSB	CM	YPD	TSB
Alginate_lyase	PIIN_08266	1.87	2.66	0.27	...	0.27	...	0.11	0.27	0.74
Alginate_lyase	PIIN_08262	0.02	...	...	...	...	...	10.96	0.44	0.47
Cellulase	PIIN_05371	0.01	...	...	...	...	...	...	...	...
Cellulase	PIIN_01558	9.04	0.27	0.01	9.41	9.14	9.05	0.23	2.20	0.16
Cellulase	PIIN_01733	...	...	...	...	...	0.07	0.04	0.04	0.13
Cellulase	PIIN_05555	1.01	0.08	...	...	...	0.09	...	0.05	0.11
DIE2_ALG10	PIIN_06071	...	...	...	...	0.11	...	...	...	...
Fn3-like, Glyco_hydro_3,	PIIN_04111	6.95	38.30	24.37	0.36	0.51	1.29	2.32	19.19	6.35
Fn3-like, Glyco_hydro_3,	PIIN_00201	0.09	...	...	...	...	0.05	0.04	0.06	0.05
GH115_C, Glyco_hydro_115	PIIN_04084	0.07	...	...	...	...	...	...	0.01	0.01
GH131_N	PIIN_01141	0.05	0.07	...	...	0.05	...	...	...	0.02
Glyco_hydro_10	PIIN_05057	...	...	...	0.01	...	...	...	...	...
Glyco_hydro_10	PIIN_04364	0.02	0.02	...	...	...	...	0.01	0.08	0.03
Glyco_hydro_16	PIIN_05222	...	...	...	...	...	...	1.78	1.84	0.71
Glyco_hydro_16	PIIN_09220	0.30	1.82	0.05	...	...	...	0.03	0.21	0.09
Glyco_hydro_16	PIIN_06486	...	...	...	0.02	...	...	0.07	0.06	0.03
Glyco_hydro_16	PIIN_00239	...	...	...	...	...	...	0.04	0.01	0.01
Glyco_hydro_20, Glycohydro_20b2	PIIN_04767	0.11	0.02	...	0.14	...	...	...	0.08	0.07
Glyco_hydro_28	PIIN_09677	...	0.05	...	...	...	...	...	0.02	0.01
Glyco_hydro_32	PIIN_08245	...	...	...	...	...	0.01	0.08	0.04	0.08
Glyco_hydro_6	PIIN_00353	0.05	0.29	0.03	...	...	...	...	...	0.01
Glyco_hydro_72, X8	PIIN_04585	0.44	...	...	0.35	0.48	0.39	0.42	0.21	0.20
Glyco_hydro_88	PIIN_01829	3.40	0.19	1.54	0.04	...	...	...	0.34	0.60
Glyco_hydro_88	PIIN_01867	0.06	...	0.01	...	...	...	...	0.02	0.01
Glyco_hydro_cc	PIIN_01066	1.17	1.03	0.72	0.55	...	0.32	...	7.85	4.65
Glyco_hydro_cc	PIIN_01067	1.81	0.68	0.18	...	...	0.22	...	5.79	2.30
Pectate_lyase_3s	PIIN_01508	...	...	...	...	...	...	...	0.01	0.02
PNGaseA	PIIN_01737	0.09	0.08	...	...	...	...	...	...	0.05
Polysacc_deac_1	PIIN_08301	0.03	0.01	0.03	0.32	0.42	1.49	0.70	0.65	1.96
Polysacc_deac_1	PIIN_05728	1.09	0.16	0.03	3.97	2.29	1.48	1.03	1.50	0.86
Polysacc_deac_1	PIIN_02364	...	...	...	...	...	...	...	...	0.01
**Subtotal (%)**		**27.69**	**45.71**	**27.24**	**15.15**	**13.27**	**14.45**	**17.87**	**40.95**	**19.73**

Supplemental Figure S2. Proteome analysis of *S. indica* EPS matrix, CW and culture filtrate. Supports Figure 2. *S. indica* spores were inoculated into three different liquid culture media: CM, YPD and TSB. The EPS matrix and CW proteins were isolated and solubilized by adding SDS-gel loading buffer and boiling at 95°C. The proteins present in the culture filtrate were precipitated using trichloroacetic acid and solubilized by boiling in SDS-gel loading buffer. The proteins were separated using a 10% SDS PAGE gel for 15 minutes and subsequently stained with Coomassie Brilliant Blue. The visible protein band in each sample was cut out and prepared for trypsin digest. The peptides were analyzed using LC-MS. Proteins with a predicted signal peptide were annotated using the protein families database Pfam. The relative abundance was calculated using the label-free quantification intensities values and expressed as a percentage (%). The proteome analysis of the EPS matrix and CW revealed that several WSC domain-containing proteins, including *Si*WSC3 (PIIN_05825), are abundant in the matrix compared to the CW. Additionally, two polysaccharide deacetylases with a LysM domain are enriched in the CW of *S. indica*. This suggests that the detected polysaccharide deacetylases with LysM domain might be involved in chitin remodeling within the CW of *S. indica.* Furthermore, a thaumatin family protein (PIIN_05281) is enriched in the culture filtrate. Thaumatins have been shown to exhibit antifungal activity through their β-1,3-glucanase activity. It is possible that PIIN_05281 exhibits an antimicrobial function against fungal competitors. Likewise, peptidases and glycosyl hydrolases with fibronectin type III module (Fn3) domains are enriched in the matrix and the culture medium but not in the CW. Fn3 domains are versatile domains in proteins that play a major role in mediating protein–protein interactions. CW: cell wall; EPS: extracellular polysaccharide; Fn3: fibronectin type III module; LysM: lysine motive; *Si*: *Serendipita indica*; WSC: cell wall integrity and stress response component.

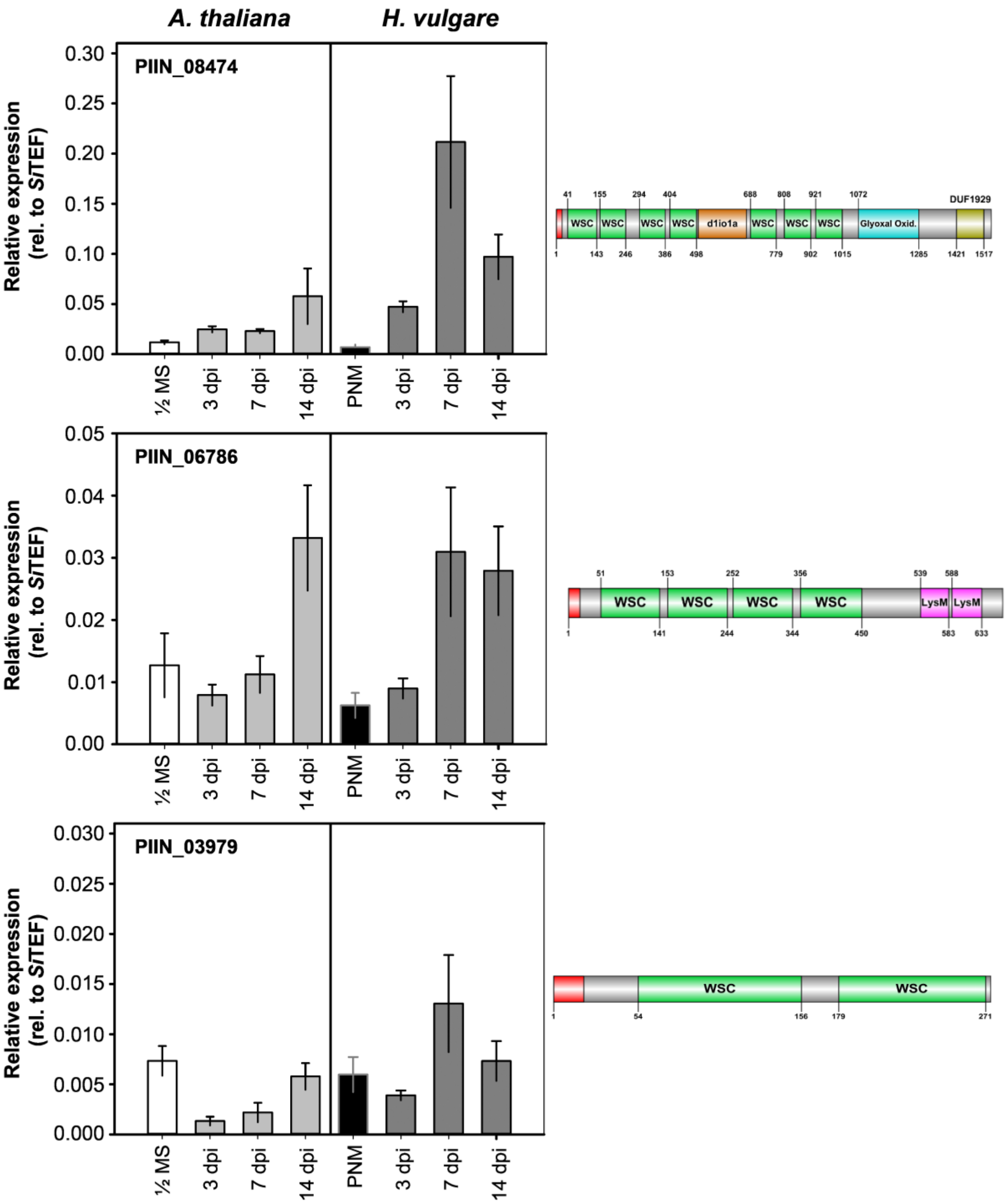

Supplemental Figure S3. Expression analysis of selected genes with WSC domains in *S. indica* during plant colonization at 3, 7 and 14 days post inoculation (dpi). Supports Figure 2. The gene expression of *S. indica* WSC domain-containing proteins during root colonization of barley and Arabidopsis was monitored via quantitative reverse transcription PCR (RT-qPCR) as previously described (Wawra et al., 2019). Average and standard deviation of three independent biological replicates (n=3) are shown. For each biological replicate, roots from several individual

plants were pooled (Arabidopsis: 60 plants; barley: 4 plants). LysM: lysine motive; MS: Murashige-Skoog medium; PNM: plant nutrition medium; WSC: cell wall integrity and stress response component.

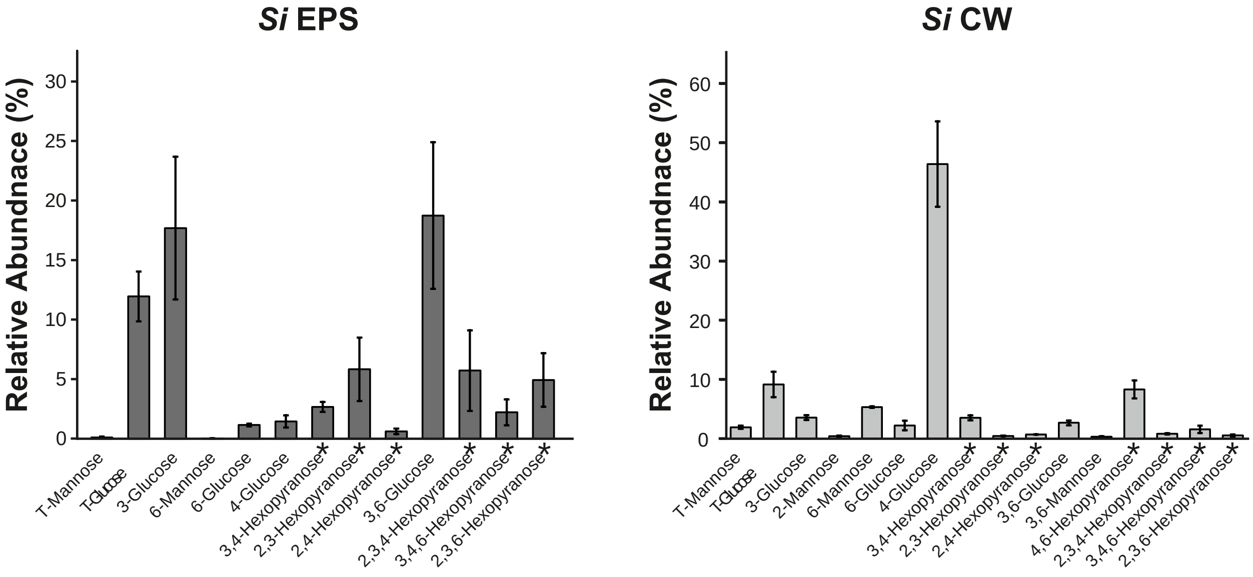

Supplemental Figure S4. Glycosyl linkage analysis of *Si* EPS and *Si* CW. Supports Figure 2. Ground EPS matrix or the alcohol insoluble residue (CW fraction) isolated from *S. indica* grown in TSB medium containing 1% sucrose was subjected to glycosyl linkage analysis as previously described (Liu et al., 2015). The sugar residues were derivatized into partially methylated alditol acetates and analyzed using GC-MS. The glycosidic linkages were assigned based on the retention time and the mass spectrum available at the publicCCRC spectral database. The annotated glycosyl residues detected in the EPS matrix or CW are expressed as a percentage of the total glycosyl peak areas. The other minor unannotated glycosyl residues are listed in Supplemental Data Set S4 and S5. Average and standard deviation of four independent biological replicates (n=4) are presented. CW: cell wall; EPS: extracellular polysaccharide matrix; *Si*: *Serendipita indica;* *exact sugar moiety unknown; overrepresentation of linkages due to undermethylation cannot be excluded.

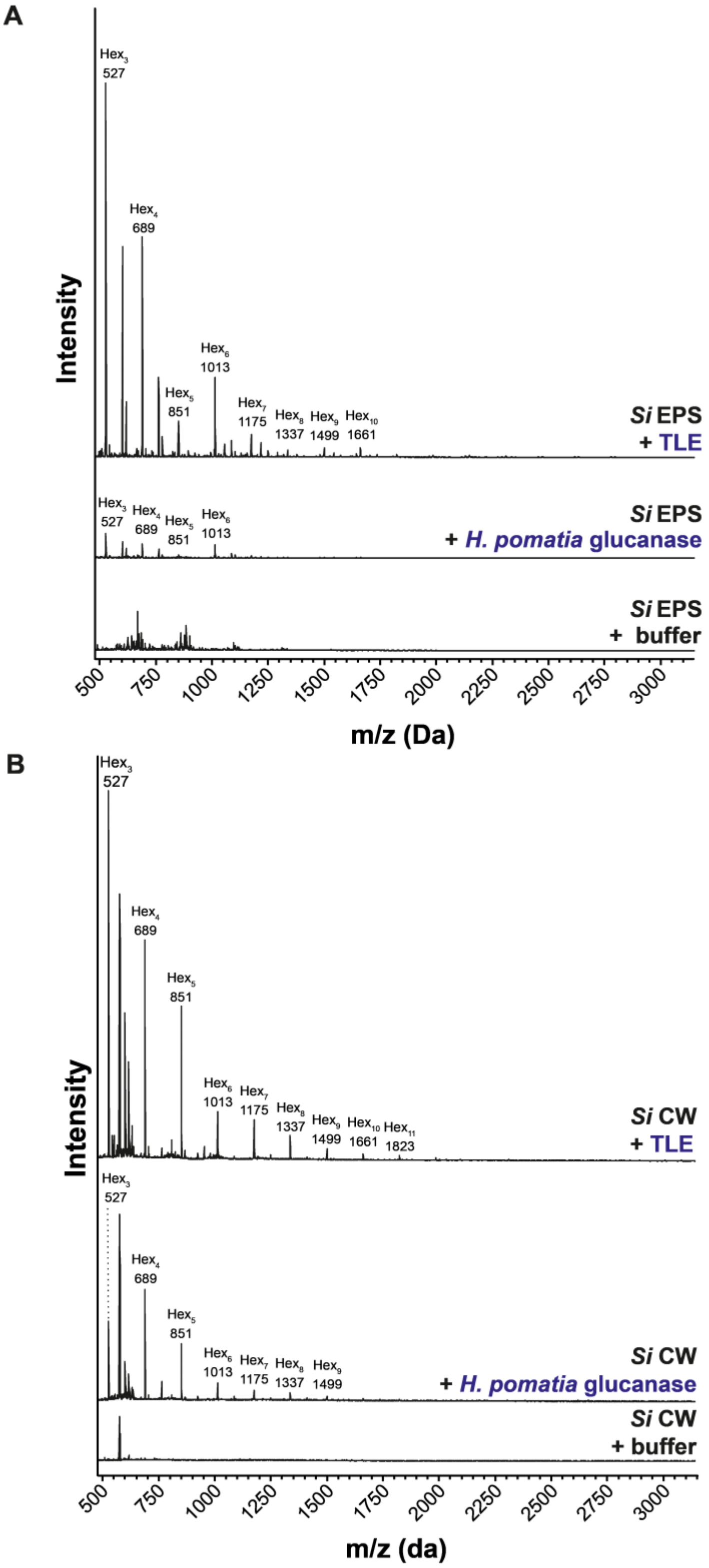

Supplemental Figure S5. Analysis of oligosaccharides released from the EPS or protein-free CW of *S. indica* by the action of β-1,3-glucanases. Supports Figure 3. 1 mg of freeze-dried EPS (A) or CW (B) obtained from *S. indica* was soaked in 2 mM sodium acetate, pH 5.0 (TLE), or 2 mM MES pH 5.0 (*H. pomatia* glucanase) at 70°C

overnight. The soaked material was incubated with TLE or *H. pomatia* β-1,3-glucanase at 40°C for 16 hours. The supernatant fraction from the digested EPS matrix and CW were analyzed by MALDI-TOF. The m/z $(M+Na)^+$ Da of oligosaccharides resulting from the digestion of the samples are labeled with their hexose composition. The digestion of *Si* EPS with TLE or *H. pomatia* glucanase was repeated independently two times with a similar result and the digestion of *Si* CW with TLE or *H. pomatia* was performed once. CW: cell wall; EPS: extracellular polysaccharide; $Hex_{n,}$: oligosaccharides with the indicated hexose composition; *Si*: *Serendipita indica*; TLE: *Trichoderma harzianum* lysing enzymes.

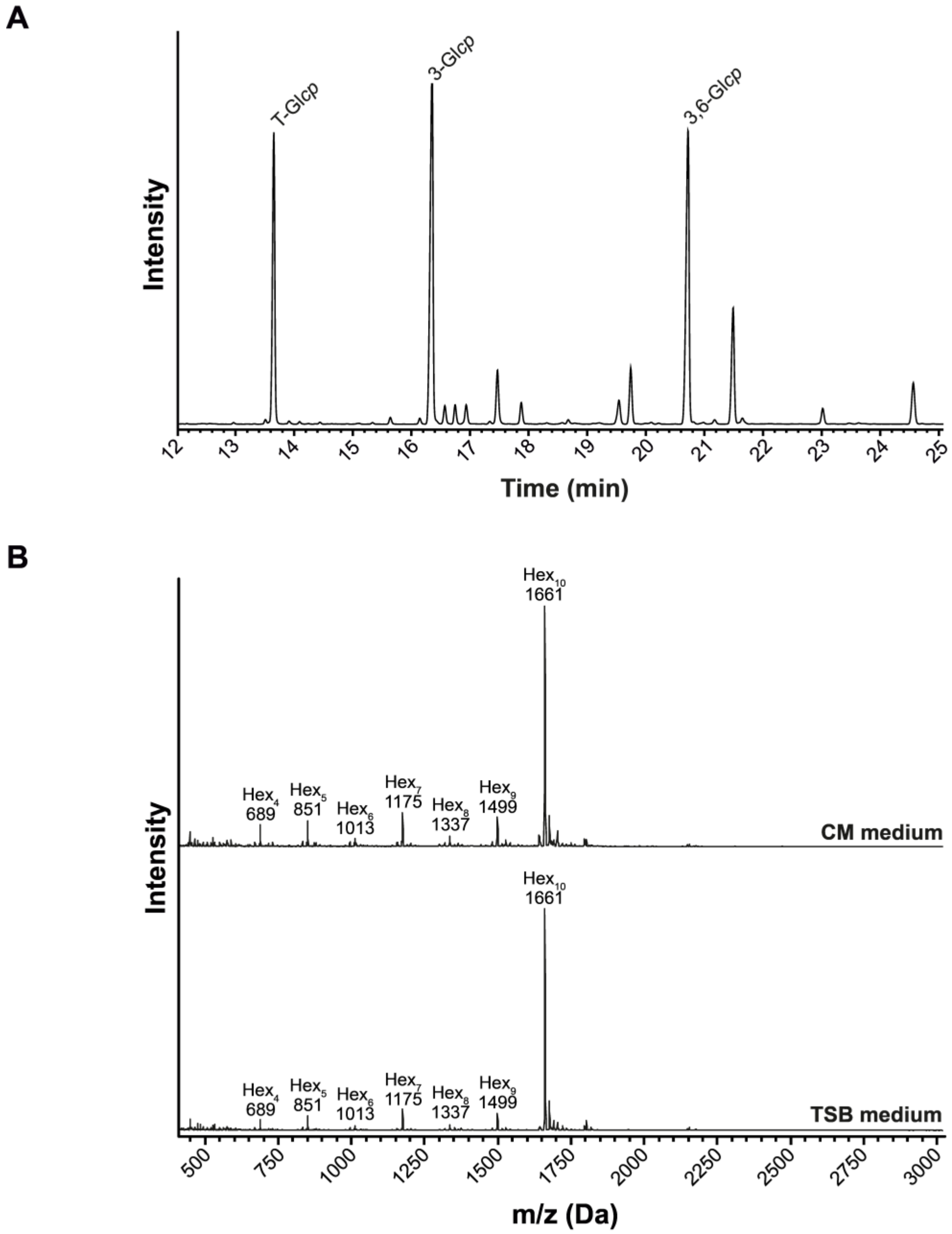

Supplemental Figure S6. Glycosyl linkage and MALDI-TOF analysis of the glucan fraction (β-GD) released from the EPS matrix of *S. indica.* Supports Figure 3. (A) Glycosyl linkage analysis of β-GD released from the EPS matrix of *S. indica*. The experiment was performed with three independent biological replicates of β-GD and the total ion chromatogram (TIC) of one of the replicates is represented. (B) *S. indica* was grown either in liquid CM or TSB medium containing 1% sucrose. β-GD was isolated and subjected to MALDI-TOF mass spectrometry. The ions corresponding to oligosaccharides with varying degree of polymerization are labelled with their hexose composition. The experiment performed with the TSB medium was

repeated independently more than three times with a similar result and the experiment performed with the CM medium was performed once. CM: complex Hill-Käfer medium; EPS: extracellular polysaccharide; Hex_n: oligosaccharides with the indicated hexose composition; TSB: Tryptic Soy Broth.

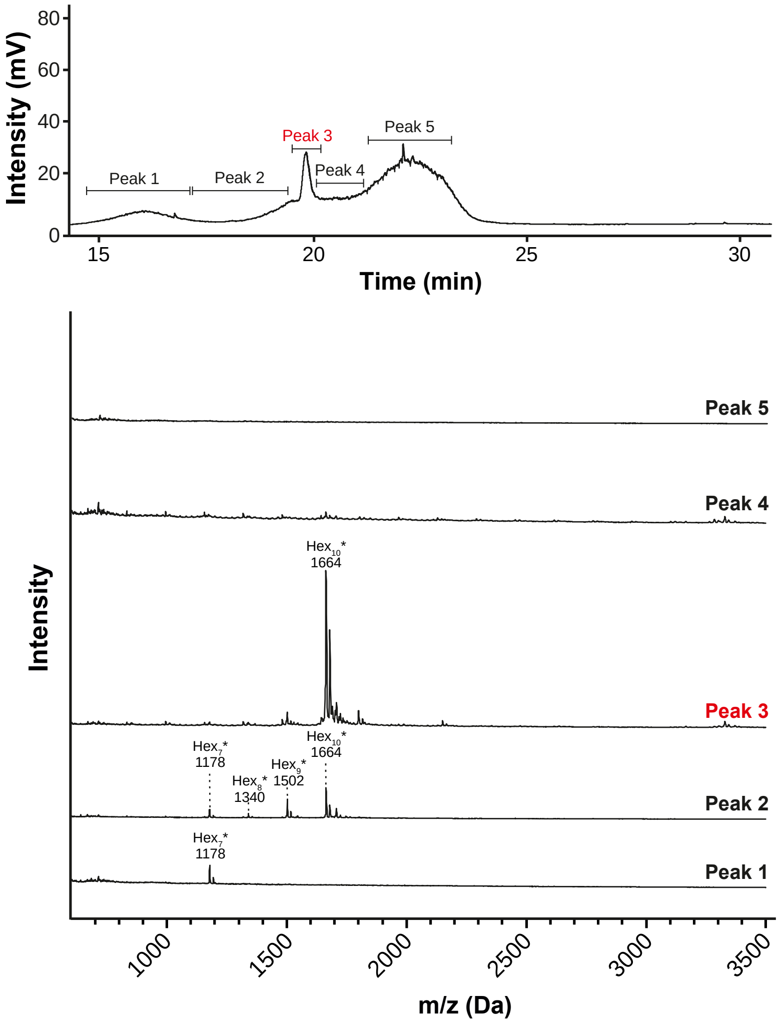

Supplemental Figure S7. Purification of the β-GD fragment for ^{1}H NMR analysis. Supports Figure 3. 40 mg of the β-GD enriched fraction was reduced using $NaBD_4$. The reduced fraction was neutralized with acetic acid, washed with methanol and air-dried. The dried material was dissolved in 2 ml of 6% methanol and 50 µl of the sample was subjected to liquid chromatography using a reverse-phase column (Vydac, Hesperia, CA, USA) connected to an evaporative light scattering detector. The detected peaks were collected and analyzed using MALDI-TOF. The samples were injected multiple times (24x) and peak 3 (shown in red) containing the reduced 1661 Da fragment was pooled and freeze-dried. The freeze-dried material was used for the ^{1}H NMR analysis. β-GD: β-1,3;1,6-glucan decasaccharide; Hex_n: oligosaccharides

with the indicated hexose composition; * represents the increase by +3 Da in the detected oligosaccharides due to the reduction of the anomeric C1 atom.

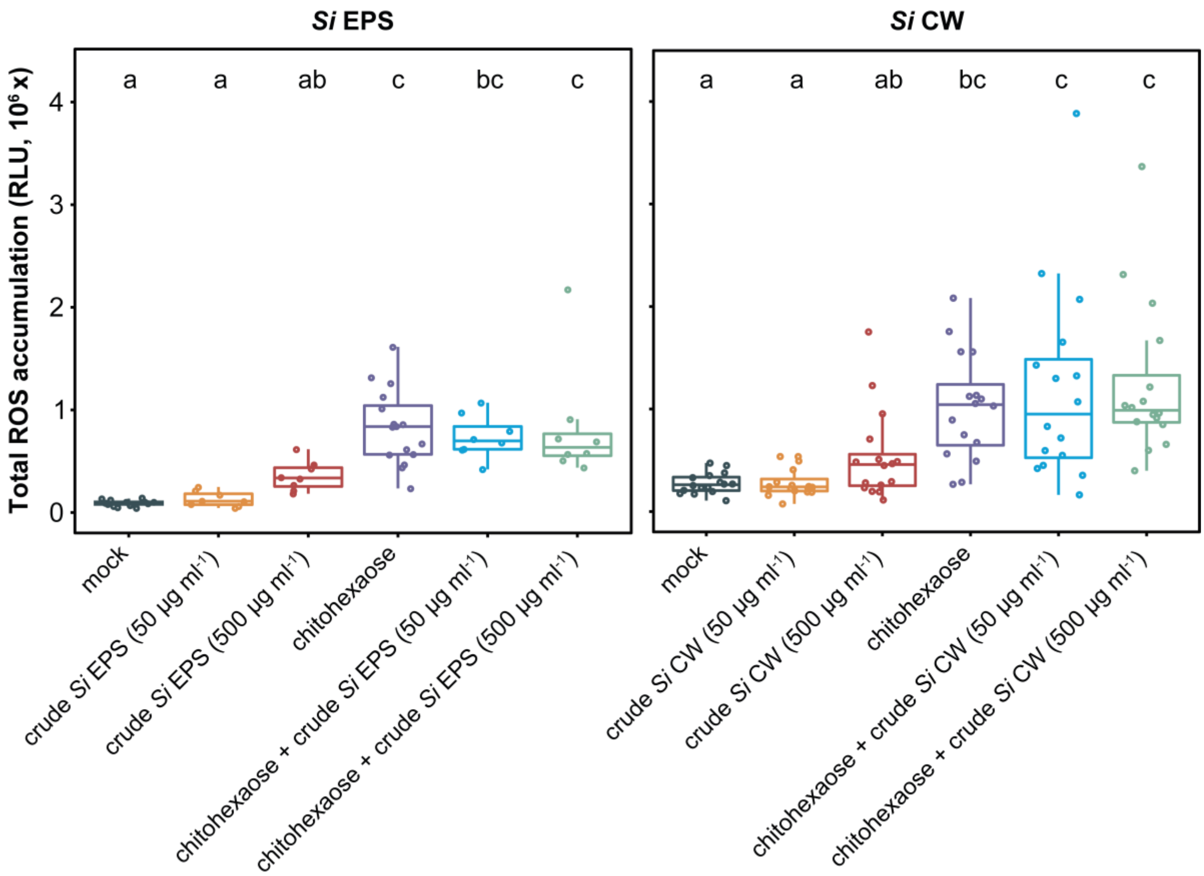

Supplemental Figure S8. Mechanically-released fragments from *S. indica* EPS matrix and CW layer do not exhibit ROS scavenging activity. Supports Figure 4. (A) *S. indica* EPS matrix and CW substrates were ground, heat-treated and sonicated prior to application to barley roots. Roots were treated with Milli-Q water (mock, n=16), chitohexaose (25 µM, n=16), crude EPS matrix or CW preparations (n=8-16) and combinations of chitohexaose and EPS matrix or CW preparations (n=8-16). Total cumulative ROS was calculated from a measured time interval of 60 minutes. Each data point in the boxplot represents the integrated value from an individual well (center line, median; box limits, upper and lower quartiles; whiskers, 1.5x interquartile range). Experiments were performed two times with similar results. Different letters indicate statistically significant differences based on a one-way ANOVA and Tukey's post-hoc test (significance threshold: p-value ≤ 0.05). CW: cell wall; EPS: extracellular polysaccharide; RLU: relative light units; ROS: reactive

oxygen species; *Si*: *Serendipita indica*.

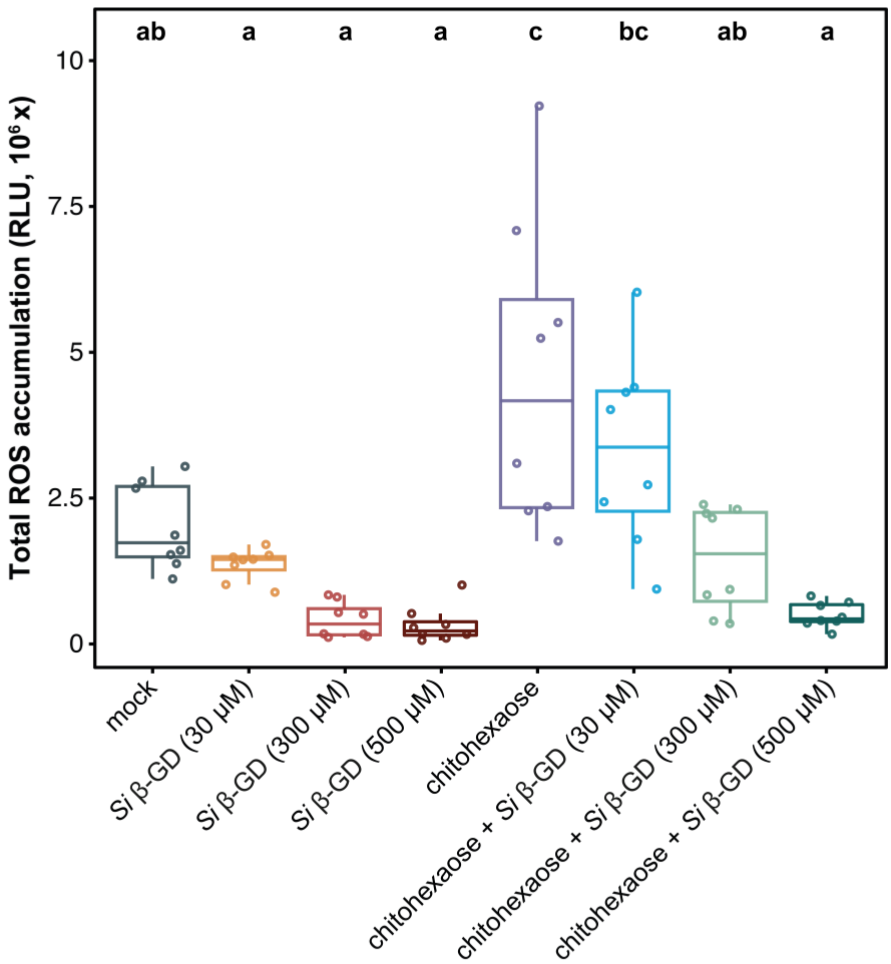

Supplemental Figure S9. Chitohexaose-triggered ROS accumulation is decreased by *S. indica* β-GD treatment in a concentration-dependent manner. Supports Figure 4. Apoplastic ROS accumulation after treatment of eight-day-old barley roots with 25 µM chitohexaose and/or purified β-GD from *S. indica.* Treatment with Milli-Q water was used as mock control. Boxplots represents total ROS accumulation over the measured time period (center line, median; box limits, upper and lower quartiles; whiskers, 1.5x interquartile range). Each data point represents measurement of an individual well containing four root pieces. In total, roots from 16 individual barley plants were used per experiment. This assay represents one out of four experiments with independent β-GD preparations. Letters represent statistically significant differences in expression based on a one-way ANOVA and Tukey's post-hoc test (significance threshold: p-value ≤ 0.05).

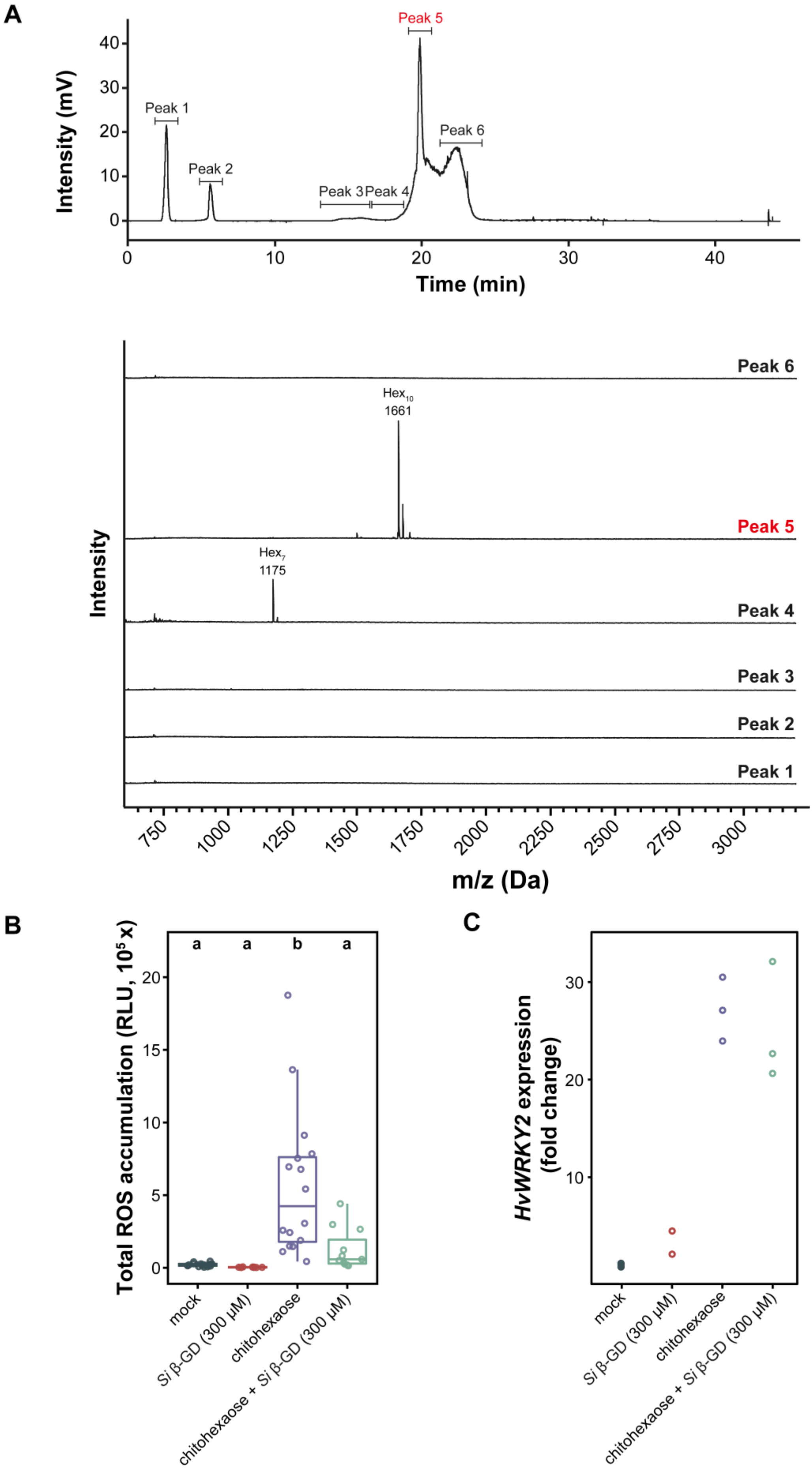
A
Intensity (mV)
Time (min)
Peak 1
Peak 2
Peak 3
Peak 4
Peak 5
Peak 6
Intensity
Hex10
1661
Hex7
1175
m/z (Da)
B
Total ROS accumulation (RLU, 10^5 x)
a
a
b
a
mock
Si β-GD (300 µM)
chitohexaose
chitohexaose + Si β-GD (300 µM)
C
HvWRKY2 expression
(fold change)

Supplemental Figure S10. Purification of native β-GD and immunogenic characterization. Supports Figure 4. (A) 40 mg of the β-GD enriched fraction was dissolved in 2 ml of 6% methanol and 50 µl of the sample was subjected to liquid chromatography using a reverse-phase column (Vydac, Hesperia, CA, USA) connected to an evaporative light scattering detector. The detected peaks were collected and analyzed using MALDI-TOF. The samples were injected multiple times (24x) and peak 5 (shown in red) containing the 1661 Da fragment was pooled and freeze-dried. HPLC-purified β-GD fraction was used in the ROS burst assay (B) and the quantitative reverse transcription PCR gene expression analysis (C) was performed using root pieces obtained from eight-day-old barley plants. (B) The following treatments were applied in the ROS burst assay: mock treatment with Milli-Q water (n=16), β-GD (300 µM, n=8), chitohexaose (25 µM, n=16) and combined β-GD/chitohexaose treatments (concentrations as in previous treatments, n=11). ROS production was measured for 25 minutes. Each data point in the boxplot represents the integrated value from an individual well (center line, median; box limits, upper and lower quartiles; whiskers, 1.5x interquartile range). Different letters indicate statistically significant differences based on a one-way ANOVA and Tukey's post-hoc test (significance threshold: p-value ≤ 0.05). (C) Samples for quantitative reverse transcription PCR were collected after 1 hour of treatment. Applied elicitor concentrations were as described above for the ROS burst assay (n=2-3). Gene expression of elicitor-responsive *HvWRKY2* was calculated relative to housekeeping gene expression (*HvUBI*) and normalized to mock treatment. Calculation of statistical significance was not performed due to low sample number. β-GD: β-1,3:1,6-glucan decasaccharide; RLU: relative light units; ROS: reactive oxygen species*; Si*: *Serendipita indica*.

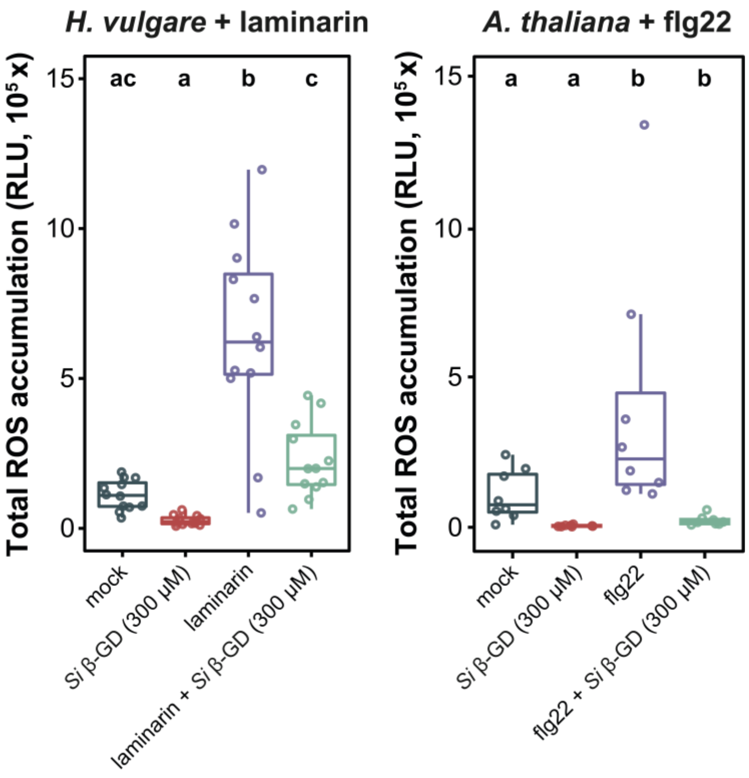

Supplemental Figure S11. Detoxification of apoplastic ROS by *S. indica* β-GD is independent of elicitor treatment and plant species. Supports Figure 4. ROS burst assays were performed with the roots of eight-day-old barley plants (n=12, data shown from one out of two independent experiments) and two-week-old Arabidopsis seedlings (n=8, single experiment). Plants were treated with Milli-Q water (mock), 2 mg/ml laminarin,100 nM flg22, 300 µM β-GD and combinations of laminarin or flg22 and β-GD. Each data point in the boxplot represents the integrated value from an individual well (center line, median; box limits, upper and lower quartiles; whiskers, 1.5x interquartile range). Different letters indicate statistically significant differences based on a one-way ANOVA and Tukey's post-hoc test (significance threshold: p-value ≤ 0.05). β-GD: β-1,3:1,6-glucan decasaccharide; RLU: relative light units; ROS: reactive oxygen species*; Si*: *Serendipita indica*.

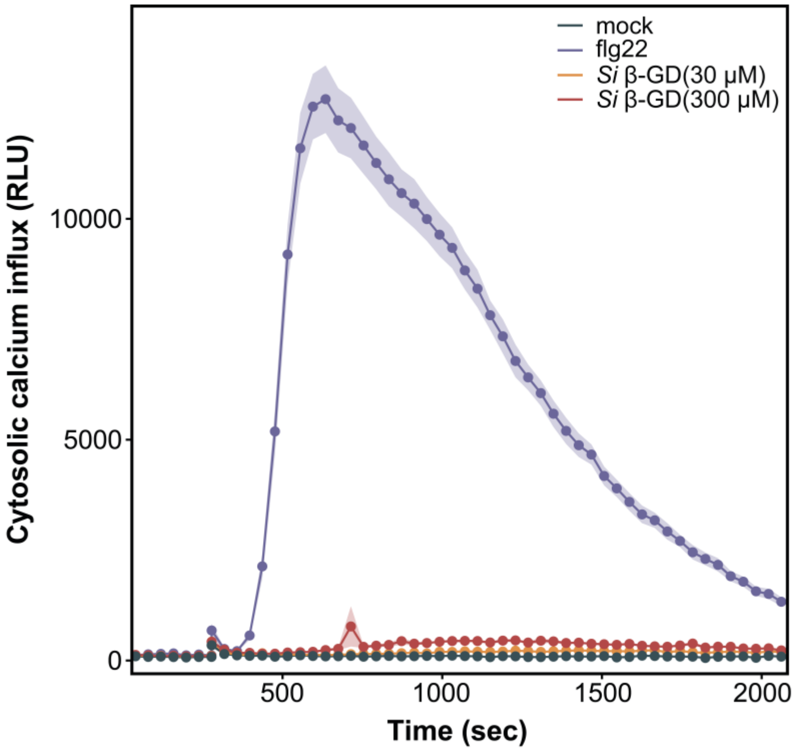

Supplemental Figure S12. *S. indica* β-GD treatment does not trigger cytosolic calcium influx in *A. thaliana* seedlings. Supports Figure 4. Calcium influx assays were performed with two-week-old A. thaliana seedlings expressing the calcium reporter protein aequorin in the cytosol. After an initial baseline measurement (5 min), seedlings were treated with Milli-Q water (mock), 100 nM flg22, 300 µM β-GD and and β-GD. β-GD: β-1,3:1,6-glucan decasaccharide; RLU: relative light units; *Si*: *Serendipita indica*. The experiment was performed once with 16 *A. thaliana* seedlings (n=16) per treatment.

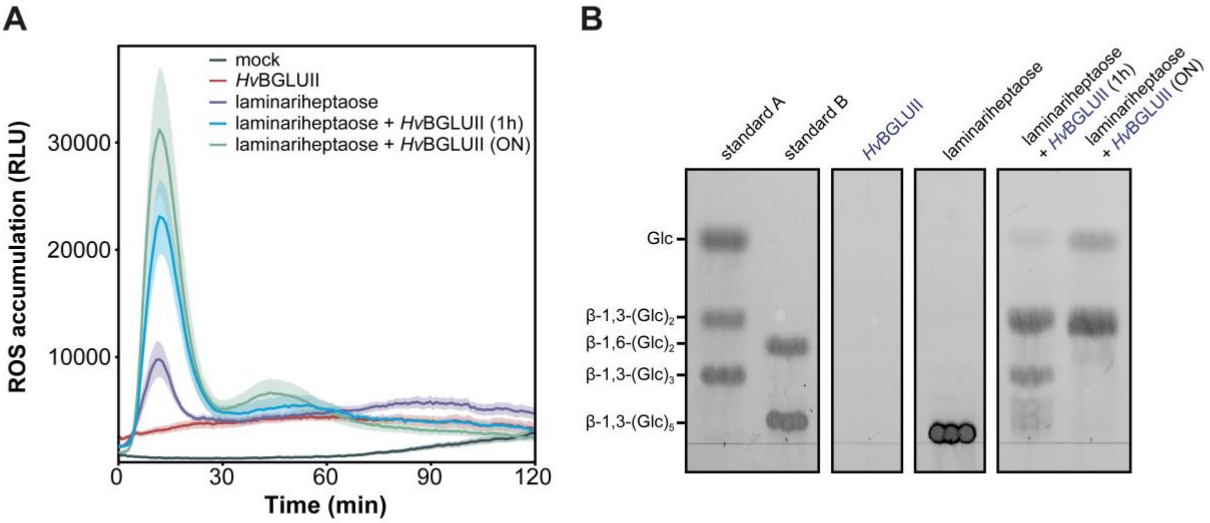

Supplemental Figure S13: Digestion of laminariheptaose with *Hv*BGLUII enhances ROS production in barley roots. Supports Figure 4. (A) Before treatment of barley roots, 3 mM laminariheptaose was digested with *Hv*BGLUII (10 units in total digestion volume of 200 µl) for one hour or overnight (ON) at 40°C, 450 rpm. As controls, Milli-Q water with *Hv*BGLUII and laminariheptaose without *Hv*BGLUII were treated in a similar way. Digestion was stopped by incubation at 90°C (10 minutes, 450 rpm) followed by centrifugation (10 min, 13000xg, room temperature). Supernatants were used in ROS burst assays with the roots of eight-day-old barley plants (n=8, three root pieces per replicate). Plants were treated with Milli-Q water (mock), Milli-Q water with *Hv*BGLUII or 300 µM laminariheptaose (digested or non-digested). (B) Digestion of substrates used in the ROS burst assay was analyzed via thin-layer chromatography. Glc: glucose; ON: overnight; RLU: relative light units; ROS: reactive oxygen species. The experiment was repeated 3 times with similar results.

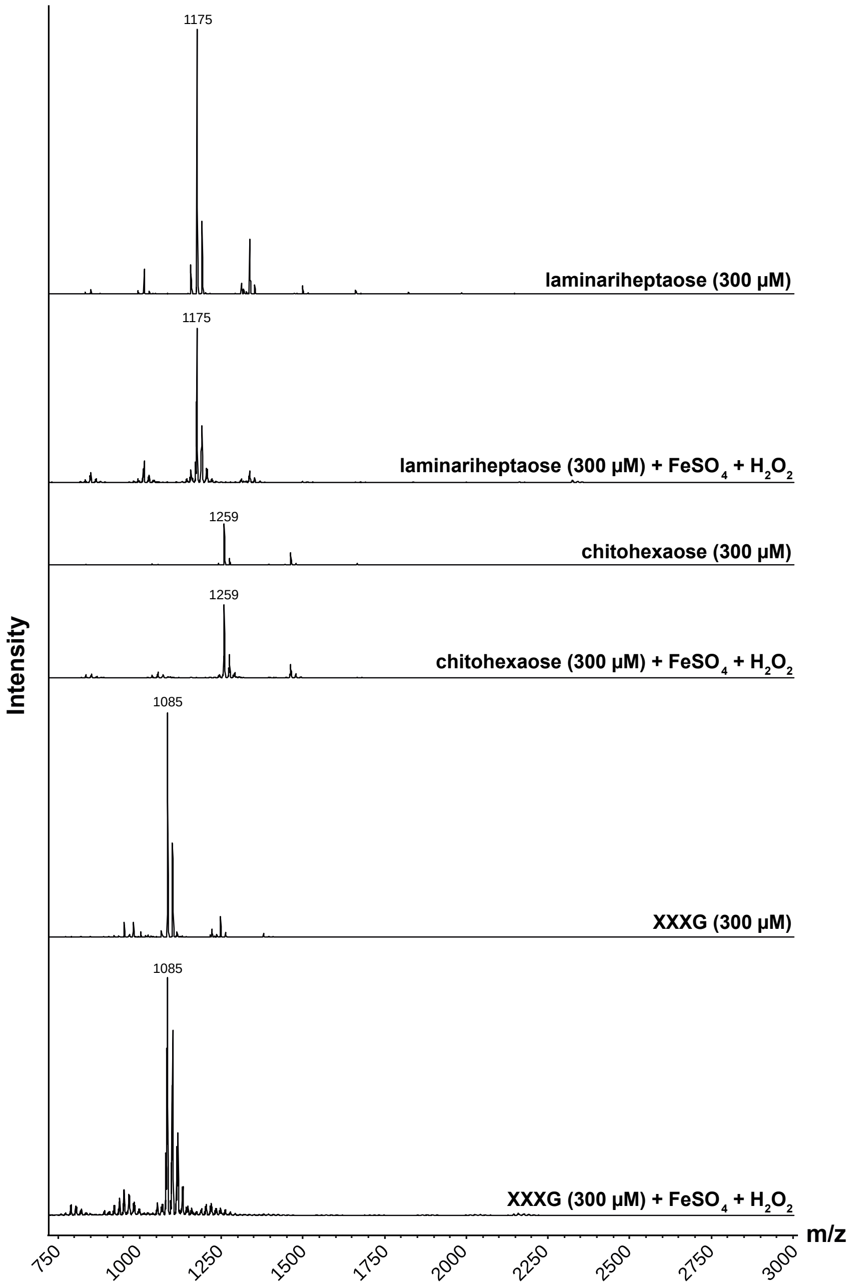

Supplemental Figure S14. Glycan controls are not degraded by hydrogen peroxide during Fenton reaction. Supports Figure 4. Laminariheptaose, chitohexaose and xyloglucan heptasaccharide (XXXG) were tested for oxidative degradation by overnight Fenton reaction (1 mM H_2O_2, 100 µM $FeSO_4$) followed by MALDI-TOF analysis. The experiment was repeated independently two times with similar results.

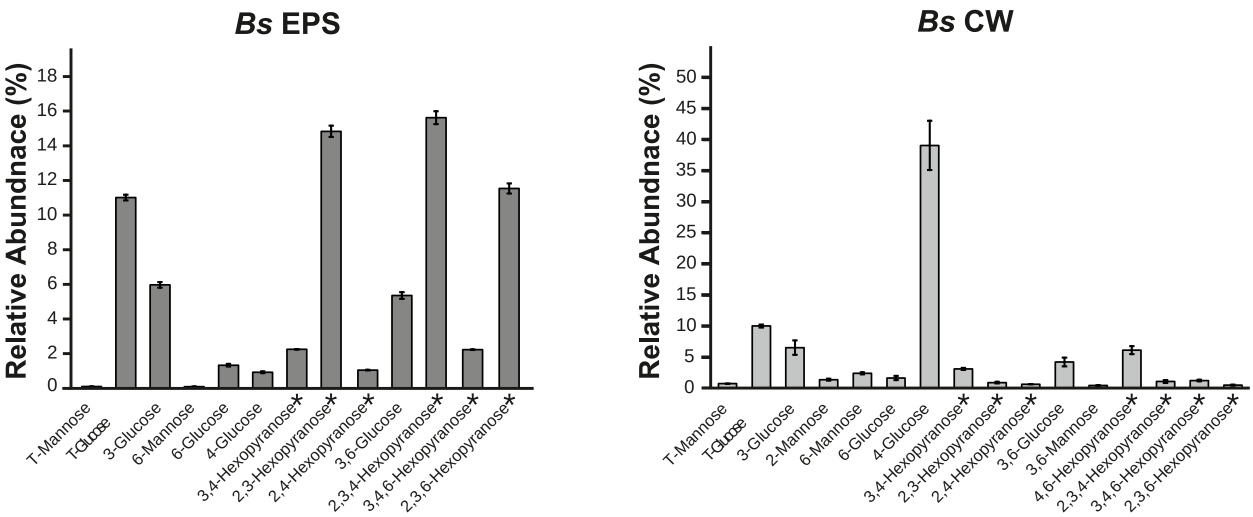

Supplemental Figure S15. Glycosyl linkage analysis of *B. sorokiniana* EPS matrix and CW. Supports Figure 5. 2 mg of ground EPS matrix or the alcohol insoluble residue (CW fraction) isolated from the cultures of *B. sorokiniana* grown in YPD medium was subjected to glycosyl linkage analysis as previously described (Liu et al., 2015). The sugar residues were derivatized into partially methylated alditol acetates and analyzed using GC-MS. The glycosidic linkages were assigned based on the retention time and the mass spectrum available at CCRC spectral database. The annotated glycosyl residues detected in the EPS matrix or CW are expressed as a percentage of total glycosyl peak areas. The other minor unannotated glycosyl residues can be found in Supplemental Data Set S7 and S8. Average and standard deviation of three independent biological replicates (n=3) are presented. *Bs*: *Bipolaris sorokiniana*; CW: cell wall; EPS: extracellular polysaccharide; *exact sugar moiety unknown; overrepresentation of linkages due to undermethylation cannot be excluded.

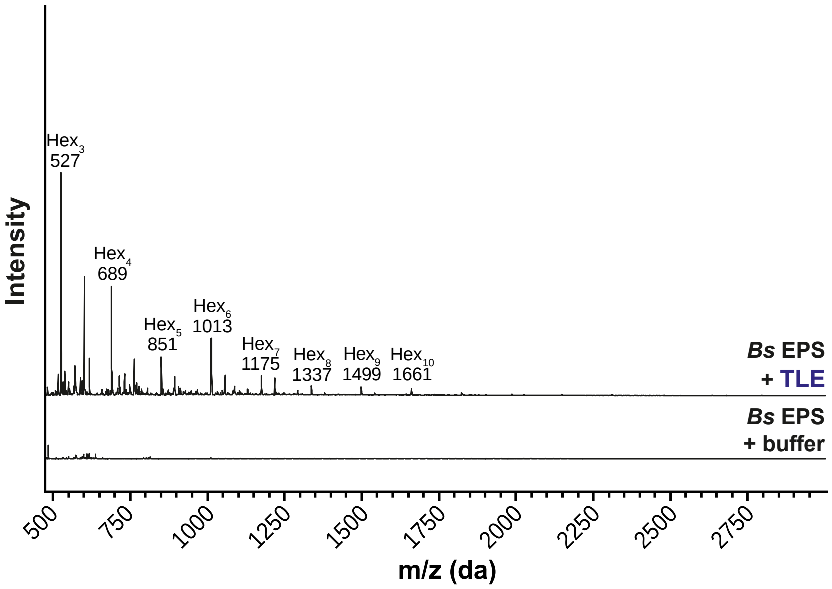

Supplemental Figure S16. Analysis of oligosaccharides released from the EPS of *B. sorokiniana* by the action of *Trichoderma harzianum* lysing enzymes. Supports Figure 5. 1 mg of freeze-dried EPS obtained from *B. sorokiniana* was soaked in 2 mM sodium acetate, pH 5.0 at 70°C overnight. The soaked material was incubated with TLE at 40°C for 16 hours. The supernatant fraction from the digested EPS matrix was analyzed by MALDI-TOF. The m/z $(M+Na)^+$ Da of oligosaccharides resulting from the digestion of EPS is indicated along with their hexose composition. The experiment was performed once. *Bs: Bipolaris sorokiniana*; EPS: extracellular polysaccharide; Hex_n: oligosaccharides with the indicated hexose composition; TLE: *Trichoderma harzianum* lysing enzymes.

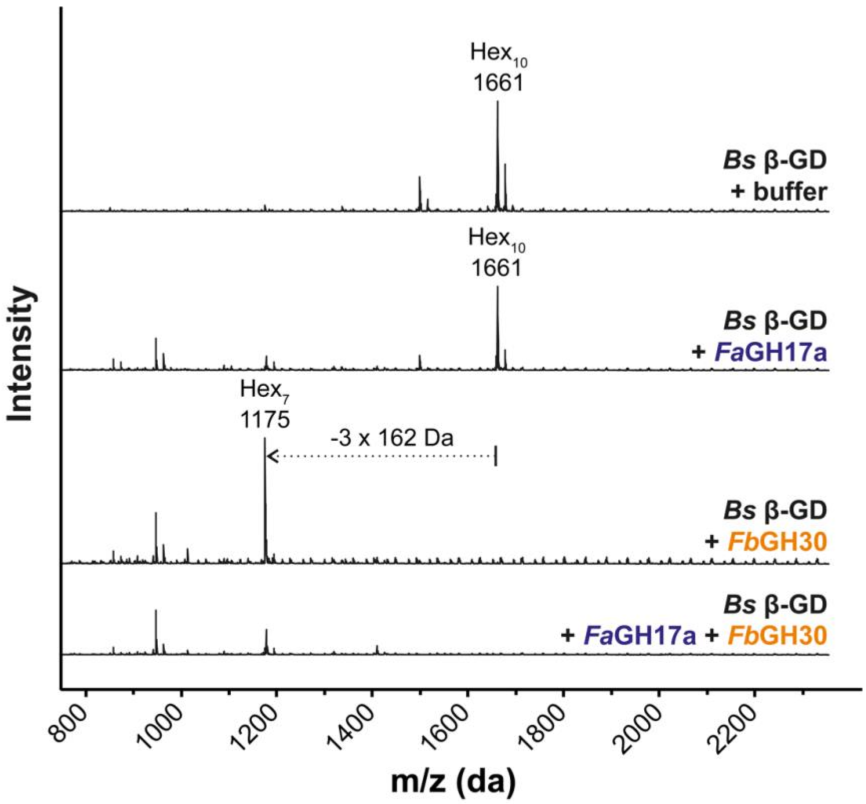

Supplemental Figure S17. The β-GD released from the EPS matrix of *B. sorokiniana* consists of a seven unit β-1,3-linked glucan backbone substituted with three β-1,6-glucosyl residues. Supports Figure 5. β-GD was treated with *Fa*GH17a and/or *Fb*GH30 at 40°C overnight. The digested β-GD fragment was analyzed by MALDI-TOF. The loss of three hexoses (-3x162 Da) as a result of *Fb*GH30 (orange) treatment is indicated with a dotted arrow. β-GD: β-1,3;1,6-glucan decasaccharide; *Bs*: *Bipolaris sorokiniana*; EPS: extracellular polysaccharide. The experiment was performed once.

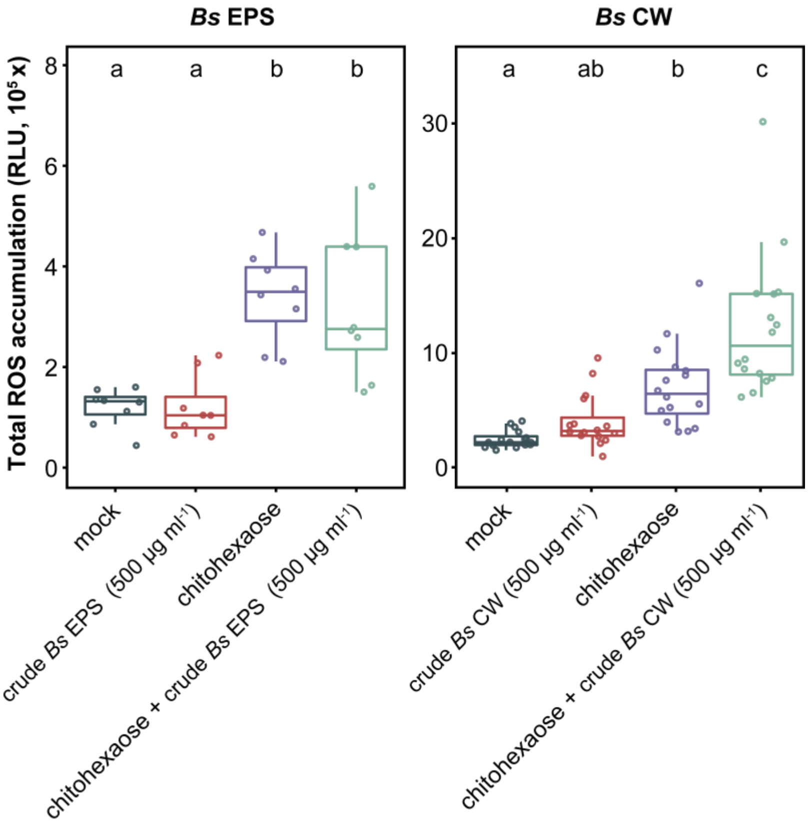

Supplemental Figure S18. Mechanically-released fragments from *B. sorokiniana* EPS matrix or CW do not scavenge ROS. Supports Figure 5. Assays were performed and analyzed as described in Figure S8. Total cumulative ROS was calculated from a measured time interval of 60 minutes. Each data point in the boxplot represents the integrated value from an individual well (center line, median; box limits, upper and lower quartiles; whiskers, 1.5x interquartile range). The experiment was performed two times with similar results. *Bs*: *Bipolaris sorokiniana*; CW: cell wall; EPS: extracellular polysaccharide; RLU: relative light units; ROS: reactive oxygen species.

Supplemental References

Liu, L., Paulitz, J., and Pauly, M. (2015). The Presence of Fucogalactoxyloglucan and Its Synthesis in Rice Indicates Conserved Functional Importance in Plants. Plant Physiology **168,** 549-560.

Wawra, S., Fesel, P., Widmer, H., Neumann, U., Lahrmann, U., Becker, S., Hehemann, J.-H., Langen, G., and Zuccaro, A. (2019). FGB1 and WSC3 are in planta-induced β-glucan-binding fungal lectins with different functions. New Phytologist **222,** 1493-1506.
