# Supplementary material for: Fungi hijack a ubiquitous plant apoplastic endoglucanase to release a ROS scavenging β-glucan decasaccharide to subvert immune responses
Source: Plant Cell. 2022 Apr 20;34(7):2765–84. doi: 10.1093/plcell/koac114 (PMC9252488; doi:10.1093/plcell/koac114)
Supplement: koac114_Supplementary_Data [file tpc21.00790.PeerReview.pdf › koac114-suppl_data/tpc21.00790.PeerReview.pdf]

# Fungi hijack a ubiquitous plant apoplastic endoglucanase to release a ROS scavenging β-glucan decasaccharide to subvert immune responses

Balakumaran Chandrasekar, Alan Wanke, Stephan Wawra, Pia Saake, Lisa Mahdi, Nyasha Charura, Miriam Neidert, Gereon Poschmann, Milena Malisic, Meik Thiele, Kai Stühler, Murali Dama, Markus Pauly, Alga Zuccaro

*Corresponding author: Alga Zuccaro (azuccaro@uni-koeln.de)*

**Review timeline:**

| | | |
|---|---|---|
| **TPC2021-RA-00790** | Submission received: | September 10, 2021 |
| | 1st Decision: | October 20, 2021 *revision requested* |
| **TPC2021-RA-00790R1** | 1st Revision received: | December 17, 2021 |
| | 2nd Decision: | January 28, 2022 *revision requested* |
| **TPC2021-RA-00790R2** | 2nd Revision received: | February 11, 2022 |
| | 3rd Decision: | February 24, 2022 *acceptance pending, sent to sci editor* |
| | Final acceptance: | March 31, 2022 |

**REPORT:** (The report shows the major requests for revision and author responses. Minor comments for revision and miscellaneous correspondence are not included. The original format may not be reflected in this compilation, but the reviewer comments and author responses are not edited, except to correct minor typographical or spelling errors that could be a source of ambiguity.)

**TPC2021-RA-00790 1st Editorial decision – *revision requested*** **October 20, 2021**

We have received reviews of your manuscript entitled "Fungi hijack a ubiquitous plant apoplastic endoglucanase to release a ROS scavenging β-glucan decasaccharide to subvert immune responses." Thank you for submitting your best work to The Plant Cell. The editorial board agrees that the work you describe is substantive, falls within the scope of the journal, and may become acceptable for publication, pending revision and potential re-review. We ask you to pay attention to the following points in preparing your revision:

- Expand the methodology section to give more details about the experimental setup.

- Clarify numbers and types of biological replicates for all experiments, and carefully describe your statistical analyses.

- Be more precise with certain statements to avoid over conclusion (see reviewers' comments).

- When B-GD can't be identified from infected/colonized barley, justify in more detail your biological hypothesis based on in vitro data.

- Include considerations based on the presence of α-glucans from starch or glycogen as contaminations in glycosidic linkage analyses

Please, keep in mind that we are reluctant to see manuscripts undergoing multiple rounds of revision and would be unlikely to offer you more than one chance to satisfy the reviewers.

--------------------------------------------------------------------------- Reviewer comments:

**TPC2021-RA-00790R1 1st Revision received** **December 17, 2021**

Reviewer comments and **author responses**:

Reviewer #1:

This manuscript reports the continuing work of the authors to characterise the gel-like extracellular polysaccharide (EPS) matrix surrounding the hyphae of different fungi during colonization of plant hosts. Previous work by the authors suggest that the secretion of soluble glycans is a common feature of plant-associated fungi.

The barley apoplastic glycoside hydrolase, β-1,3-endoglucanase HvBGLUII, is shown to release a conserved β-1,3;1,6-glucan decasaccharide (β-GD) from the EPS matrices of two fungi i.e. the root endophyte Serendipita indica (Basidiomycota) and the pathogen *Bipolaris sorokiniana*.

Point 1. The title of this manuscript is: "Fungi hijack a plant apoplastic endoglucanase to release a ROS scavenging β-glucan decasaccharide to subvert immune responses". Unfortunately, I could not find any details of the ROS burst assays in the Supplemental information describing the Materials and Methods used in the studies.

> **RESPONSE: The experimental details of the ROS assay are described in the SI Materials and Methods file, within the Plant immunity assays section. This part of the method section has been restructured to improve the clarity of this paragraph.**

Point 2. Lines 205-222 and Figure S8 describe the data obtained using assays in which mechanically-released fragments from the S. indica EPS matrix and CW were applied to barley roots. Roots were treated with chitohexaose, crude EPS matrix or CW preparations and combinations of chitohexaose and EPS matrix or CW preparations.

The authors should be more careful with regard to wording and avoid exaggeration. For example, the data shown in Figures S8, S9 and 4 reflect ROS accumulation and not ROS production, as stated in the text.

> **RESPONSE: We thank the reviewer for the remark. The term "ROS production" was changed to "ROS accumulation" in the text and figures.**

Point 3. Line 211 states that "Remarkably, applications of the β-GD led to significantly lower ROS levels compared to the mock treatment (Figure 4A)". However, the data shown in Figure 4A are far from convincing, particularly as there are no statistics on these data. The results shown in the other parts of figure 4 and Figures S9 and Figure S10 regarding the combined application of chitohexaose and β214 GD (Figure S9) are clearer. However, the statement that "decreased accumulation of ROS with increasing concentrations of added β-GD is not evident from the data shown in Figure 4A or Figure S9. As far as I can see, only two levels of β-GD were added.

> **RESPONSE: The statistics of the presented line graphs are performed on the curve integrals, which are presented in the boxplot on the right side of the graph in Figure 4A (total ROS accumulation).**
>
> **Additionally, we have included a new Supplementary figure (Figure S10) which presents a wider range of applied β-GD concentrations to corroborate our conclusion in this text passage.**

Point 4. The statement that "Combined digestion of the β-GD with the endoglucanases FaGH17a and FbGH30 restored the chitohexaose-triggered ROS burst (Figure 4B)", refers to a conclusion and not a result. However, it is clear that decreased apoplastic ROS accumulation is linked to the presence of intact β-GD. Statements suggesting that "β-GD significantly reduced ROS bursts in barley roots", are misleading because they could infer that β-GD impairs ROS production by the RBOH or similar enzymes.

> **RESPONSE: We agree, this could be misleading and it is not what we want to imply. To stay in line with the reviewer's comments above, we modified the mentioned text passage to "β-GD significantly reduced apoplastic ROS accumulation in barley roots".**

Point 5. The fact that β-1,6-linked glucose branches and β-GD can scavenge ROS is interesting and important but it should be remembered that such antioxidants are sacrificial because the reduced forms cannot be regenerated. Hence, the antioxidant value of β-GD is strictly "single use", and hence the β-GD pool may be easily depleted in planta. This fact should at least be mentioned in the discussion section. The ROS scavenging properties of soluble β-glucans may well be important in host colonization but a temporary block that limits the extent of the oxidative burst may be all that is required.

> **RESPONSE: We thank the reviewer for the suggestion. We agree and have added a sentence on the topic of single**

**use of the β-GD to the discussion.**

Reviewer #2:

The manuscript of Chandrasekar et al shows the existence of a yet undescribed strategy used by both fungal endophytes and parasites to cope with plant immune defenses. Such mechanism is based on the selective release of a B-glucan decasaccharide (B-GD) with ROS scavenging activity that the authors partially characterize at a structural level.

The authors have performed a large, well-designed, and comprehensive study to tackle the mechanistic modes of action of B-GD from its release from fungal EPS matrix to its ROS scavenging activity both in vitro and on plant tissues. They also show, using diverse biochemical approaches, the unique properties of B-GD in respect to other already described immunogenic B-glucan oligomers usually released during plant infection laying the basis for the understanding of how different glucan-based molecules can be used in nature for different purposes.

The story is well written and keeps the reader engaged to guess what's next. Overall, the paper is of high scientific quality, however, I see few critical issues preventing the publication of this manuscript in its present form.

Point 1. The authors are able to extract and characterize B-GD from enzymatic digestion of EPS with the barley β-1,3-endoglucanase HvBGLUII, however, it is not clear if the same happens in vivo in the onset of fungal invasion of plant tissues. In my opinion, the authors should be able to identify B-GD from infected/colonized barley roots to make sure that their biological hypothesis is true.

**RESPONSE: Our confocal microscopy data together with the apoplastic proteomic dataset show that EPS matrix and HvBLUII overlap during fungal colonization, both in a spatially and timely manner. We very much agree that it would be desirable to identify the β-GD in vivo. However, due to analytical constrains, this is unfortunately extremely difficult. Also, we agree that based on our results the β-GD is "single use" and hence would not accumulate in an amount which would be sufficient for detection in planta.**

**To test this we incubated the purified fragment with apoplastic fluids from mock-treated barley roots and roots challenged with the two fungi. Whereas the fragment remained intact when treated with the mock-treated apoplastic fluid (in accordance with its structure-dependent resilience), its amount decreased within 1 hour of incubation and disappeared overnight in combination with the activated apoplastic fluids. These activated apoplastic fluids presented an increased oxidative potential compared to the mock-treated samples, measured by DAB assay, which is indicative of higher ROS levels in these samples. These data are in agreement with our hypothesis and we are aware that this does not represent final proof but it shows that at this point we have no easy way to analyze this in planta.**

Point 2. Second, it is not always clear what experimental setup was used to obtain the data sets shown in individual figures. In general, the methods used are described either referring to previous articles or with minimum details (see for example the confocal microscopy section of the Supporting information). This lack of details in the methods usually makes it difficult for the reader to understand to which figure panel they refer to.

**RESPONSE: Missing information was added in M&M and in the legends.**

Point 3. Additionally, information on independent experiments and the number of replicates should be added. In some figure legends (figure 1, 2C,3B-E, 5, S3, S4, S5, S6, S12-15) information on how many independent experiments were performed and how many replicates per treatment were used is completely lacking.

**RESPONSE: Missing information wasadded in M&M and in the legends.**

Point 4. In figure S3 the authors show gene expression after 7 and 14 dpi, however in the methods, they say to have harvested plant material only after 3 dpi. Is that an error or there is a missing methods paragraph regarding this experiment?

**RESPONSE: In this figure we show data for 3, 7 and 14dpi the methods referred to the colonization with S. indica. More info was added in the legends and M&M.**

Point 5. In general, a very careful reading and editing of the methods section and figure legends should be performed by the authors to clarify the above-mentioned issues (2-4) before publication.

**RESPONSE: We thank the reviewer for their comment, the mentioned sections were revisited and edited.**

Reviewer #3:

The work of Chandrasekar et al attempts to deepen the understanding of plant glycan-triggered immunity, which probably due to its technical complexity has not advanced much in the last 30 years. The work is conceptually very interesting, as it proposes the possibility that certain fungal-derived glycans could act as inhibitors of ROS production by plants. Indeed, some parts of the work are brilliant. However, I found several problems throughout the manuscript that should be clarified before this interesting story could be published.

Point 1. One of the main problems is that the glycosidic linkage analyses have been performed on materials that most likely contain α-glucans from starch or glycogen, which are common contaminations from the media or the fungal intracellular storage polysaccharides. This causes that, especially in the CW fractions, the main component is 4-Glc, probably α-linked and not β as proposed in line 145. In principle this would not affect the subsequent purification of β-GD, but it certainly invalidates the results of figures 2C, 5A and S4. In this regard, the authors indicate that they assign 85% of the glycosidic linkages (L 140), but I wonder why they have not used a large library of oligosaccharides as standards to identify 100% of the signals, which is perfectly possible nowadays. Finally, the presence of 2,3-hexose, 2,3,4-hexose and 2,3,6-hexose (approx. 40%) in the B. sorokiniana EPS matrix should be explained, as it seems to indicate possible undermethylation events. All these aspects should be considered by the authors.

**RESPONSE: The material has been digested with an α-amylase to test for any potential glycogen/starch contaminants (see new Figure S5). However, only minor amounts of carbohydrates were released upon amylase digestion without showing the expected glycogen/starch digestion profile (Figure S5). Hence, the 4-Glc present (e.g. dominantly in the CW fraction) does not represent glycogen but likely a cellulosic-based glucan material or α-glucan specific to the cell wall. We discussed this possibility in the new version of the manuscript.**

**Concerning the assignment of the glycosidic linkages: During the derivatization of materials to partially methylated alditol acetates other non-carbohydrate components (e.g. lipids or secondary metabolites) are also derivatized that will result in additional peaks in the following GC-chromatograms. The mass spec fragmentation pattern of these peaks clearly indicates non-carbohydrate components. Therefore, these 15% of the signals were not assigned, but of course their presence needs to be mentioned.**

**Undermethylation can always occur during the derivatization procedure due to the presence of precipitated/ semi-crystalline material of the material (in particular the cell wall material) and sometimes limited access of the reagents. Undermethylation cannot be excluded, but the results are quite clear when it comes to the differences between the EPS and CW fraction. While the mass fragmentation pattern clearly indicated a 2,4-hexose, 2,3-hexose etc. the exact nature of this hexosyl-moiety (glucose, mannose, or galactose) could not be ascertained as a GC retention time of the corresponding derivatives was not available. Based on the prevalence of the monosaccharides in the fungal extracellular matrix these derivatives likely represent glucosyl-moieties. These issues are now explained in the legend of Figure S4.**

Point 2. In recent years, several glucans (MAMPs or DAMPs) have been identified, which trigger PTI responses in plants, and even protect from infection against pathogens, but they generally do so in a ROS-independent manner (e.g. cellobiose). In this work authors did not find ROS production by β-GD, but an increase in HvWRKY2 expression. I wonder what would happen if additional PTI hallmarks to ROS were analysed in barley or Arabidopsis (e.g. MAPK phosphorylation or calcium influxes). Would it be possible that β-GD would trigger them, and the ROS inhibition would not be unique to β-GD, but to other glucans? I think it would be key to delve deeper into this type of experiments to be able to ascertain the role of β-GD.

**RESPONSE: We thank the reviewer for the excellent suggestion. While dissecting the entire suite of immune responses would be very informative, the isolation of β-GD from the fungal EPS layer leads to restricted amounts of substrate. Since many typical PTI assays need substrates amounts in the milliliter range, this prevents us from including a wide range of PTI assays for all the different approaches (β-GD digest, co-treatments with chitohexaose etc.). To address the question whether treatment with β-GD other hallmarks of plant immune responses, we added an additional figure where we show that β-GD does not lead to cytosolic calcium influx in Arabidopsis seedlings (Figure S13).**

Point 3. Figures 1 & 2; this part of the paper is rather disconnected from the rest and from the main finding, and in my opinion hinders the interpretation of the subsequent results. I do not completely agree that the results in Fig 1 show that the EPS matrix is particularly enriched in β-1,3-glucans or at least do not exclude that the CW would also. The proteomic study is interesting, but I don't see that it adds much to the body of the article. Regarding the proteomics, I have not been able to find the statistical details, especially about the number of independent experiments.

**RESPONSE: In principle, we agree with the reviewer but after thorough consideration we concluded that these 2 figures are sufficiently linked to the story. In figure 1 we show the presence of the matrix in planta and we use SiWSC3 as probe for beta 1,3-glucans in vivo corroborating our proteomics and glycomics data. We do not claim that this is the sole glucan present and we do not exclude that the CW would also have beta-glucans.**

**Regarding the proteomics, these are 3 independent experiments performed in 3 different media. If a protein is represented (and abundant) in a specific compartment in all 3 media we can exclude a medium effect. The analyses are based on normalized LFQ (label-free quantitation) intensities (quantitative proteomics). The protein intensities were normalized to exclude some "outliers" to best represent the ratio changes of different samples and represents real abundances (columns AS to BA in Table S1). Higher intensity means higher abundance of a specific protein. This is represented in percentage in the figure 2 and S2. We better stated this in the new version. Additionally, we included an annotation enrichment analysis to support the claim that WSC domain containing proteins are higher abundant in the EPS matrix and supplied a statistical analysis in a new supplemental table S3.**

**TPC2021-RA-00790R1 2nd Editorial decision – *revision requested* January 28, 2022**

We have received reviews of your manuscript entitled "Fungi hijack a ubiquitous plant apoplastic endoglucanase to release a ROS scavenging β-glucan decasaccharide to subvert immune responses." Thank you for submitting your best work to The Plant Cell. The editorial board agrees that the work you describe is substantive, falls within the scope of the journal, and may become acceptable for publication, pending revision, and potential re-review.

We ask you to pay attention to the comments of Reviewers #1, and #3 and those from the Reviewing Editor while preparing your revision.

**TPC2021-RA-00790R2 2nd Revision received February 11, 2022**

Reviewer comments and **author responses**:

Reviewer #1:

This manuscript describes new and interesting information concerning the role of the barley (β-1,3-endoglucanase HvBGLUII) that releases a conserve 1,3;1,6-glucan decasaccharide (β-GD) from the EPS matrices of fungi.

Point 1. While I can see that improvements have been made in revision of this manuscript, the interpretation of the data relies on the accurate estimation of changes in reactive oxygen species (ROS) levels. I cannot see how the methods used preserve any ROS generated in the system, or provide any accurate or any unambiguous information concerning changes in ROS levels. Perhaps I have misunderstood the ROS assay methods that were used and hence a much clear explanation is required. The details given are too brief for clarity and give no indication of exactly what was measured.

For the ROS assay, well plates with plant material were incubated overnight at room temperature on the bench. ROS are extremely susceptible to metabolism or simply removal by enzymes in the extracellular matrix. Why was incubation overnight performed and at room temperature? This makes no sense. Any hydrogen peroxide formed will have rapidly been metabolised. The next day water was replaced by 100 µl of fresh Milli-Q water containing 20 µg/ml horseradish peroxidase (Sigma-Aldrich, Taufkirchen, Germany) and 20 µM L-012 (Wako Chemicals, Neuss, Germany). How can horseradish peroxidase function in water, without any pH buffering? After 15 minutes incubation elicitor solutions were added to the wells and measurements were made. However, I am not clear what was being measured. Definitive proof must be provided that the methods used accurately measure and preserve ROS.

**RESPONSE: We thank Reviewer 1 for the critical comments on our approach to quantify apoplastic ROS production**

**upon elicitor treatment and to give us the possibility to improve the material and methods with a detailed explanation of this standard method.**

**Here a few explanatory comments to clarify the raised questions: Upon binding of immunogenic substances by membrane-integral receptor kinase complexes (e.g. fungal chitin / glucan or the bacterial flagellin epitope flg22), plants initiate transient apoplastic ROS production within only a few minutes. The ROS burst assay used in this manuscript is based on the widely used, standard procedure to measure these elicitor-triggered apoplastic ROS bursts; the principle has not been changed over the last 20 years (Felix et al., 1999; Ngou et al. 2021). The overnight incubation step of plant tissue (leaf or root pieces) in water aims at removing components (e.g. ROS) released as consequence of the mechanical damage (i.e. cutting the tissue). On the next day, the water was exchanged for an HRP/L-012 solution. To not interfere with plant immune responses downstream of localised proton fluxes, this solution is not provided in buffer but instead in MilliQ water (pH 7-7.5). In fact, buffering in the acidic range (pH 5.5-6.5) has been shown to compromise elicitor-triggered immune responses such as cytosolic calcium influx, ROS production and PR gene expression (Wang et al., 2018; Albert et al., 2017). After a short incubation time to reduce the background noise, the elicitor solutions were added and the elicitor-triggered ROS production was immediately measured. All added elicitors solutions (water as mock control, chitohexaose, flg22, decasaccharide, enzymatically degraded decasaccharide and their respective combinations) had the same pH (~pH 7.5), hence it can be excluded that the observed differences in ROS production are due to pH differences. After the addition of the elicitors, the HRP immediately catalyses the oxidation of L-012 (luminol derivate) by the produced ROS (mainly hydrogen peroxide derived from superoxide anions). This chemical conversion results in a chemiluminescent reaction which can be quantified by the plate reader.**

**Since the pH of the decasaccharide wasn't different from the other elicitor solutions and enzymatic degradation of the decasaccharide abolished the observed ROS scavenging, we are convinced that the observed data is real and not a technical artefact. In addition to the HRP-based ROS production assays with plant tissues, the DAB assay (HRP-based, buffered with 50 mM sodium acetate at pH 5) and the Fenton reaction-based oxidation of sugars (HRP-independent, no buffer) corroborate our observation that the purified decasaccharide has the potential to affect ROS.**

**To facilitate the understanding of the applied method, we have modified the ROS burst assay description in our materials & methods section.**

<u>Reviewer #2:</u>

I have just a few minor comments on the text which I believe are necessary to improve the manuscript:

Point 1. Number of independent experiments and replicates is still missing for confocal datasets.

**RESPONSE: The confocal microscopy was repeated several times with 2 independent SiWSC3-His-FITC488 batches and colonized plants. The missing information was added to the legend of figure 1. The observed fungal matrix was not a sporadic observation but regularly observed with both fungi.**

Point 2. I'm still not sure to have understood to which exact method figure S3 relates. If it relates to plant immunity, then 3 dpi is missing in the methods text. Nevertheless, this point should be clarified, and the readers should readily understand which methods have been used for each dataset. Please make it clearer. Also, what does (n=3) means in the figure S3 legend? Independent experiments, replicates? This should be stated in both methods and legends. Please, revise very carefully both the methods section and figure legends to include such details which are absolutely mandatory.

**RESPONSE: We have modified the legend of figure S3 and the methods section. Figure S3 is a kinetic of S. indica colonized roots at 3, 7 and 14 dpi.**

Reviewer #3:

I am grateful to the authors for trying to answer most of the questions that the previous version of the manuscript had raised.

Point 1. However, those related to the data shown in figures 2C, 5A, S4, S5 and S16 are, for me, still not fully answered. I find it surprising that the authors have used state-of-the-art technologies in glycan characterisation (mass spectrometry, NMR...) and intend to solve this problem with a TLC. For me, the necessary experiment would be to do the linkage analysis of the corresponding fractions after the enzymatic digestions and show that the 4-glc peak is still there. As I indicated in my comments on the previous version, this does not invalidate the subsequent conclusions, but if not correct, it may be confusing for the scientific community, since such high levels of beta-1,4-glucans have not been previously demonstrated in fungi.

Point 2. On the other hand, the answer to the problems of "undermethylation" is also unsatisfactory. According to the authors, the problem could be that they are not "glucosyl moieties" and they solve it with an asterisk, but in reality they should change it to "hexopyranoses". Moreover, the problem with undermethylation would not be that, but the incorrect assignment of bonds, since it would indicate more glycosidic bonds than actually appear in the molecule before methylation. To my knowledge, bonds in position 2 are quite strange in fungal glucans, and I think this is something that it would be interesting to firmly demonstrate or eliminate from the manuscript.

> **RESPONSE: We agree with both points 1 and 2 raised by this reviewer and in agreement with the editor´s comments we reformulated the relevant part in the text to state that our data are not conclusive to affirm that the glucans found in the fungal CWs are beta-1,4-glucans as they can come from mixed-linked glucans and that several of the links are of doubtful identification. We also removed the TLC figure from supplementary part. We modified the legend of figure 5 and figure S4 and S15 (old figure S16) by changing glucose to Hexp and by pointing out in the legend that *exact sugar moiety unknown; overrepresentation of linkages due to undermethylation cannot be excluded.**

---

**TPC2021-RA-00790R2 3rd Editorial decision – *acceptance pending* February 24, 2022**

---

We are pleased to inform you that your paper entitled "Fungi hijack a ubiquitous plant apoplastic endoglucanase to release a ROS scavenging β-glucan decasaccharide to subvert immune responses" has been accepted for publication in The Plant Cell, pending a final minor editorial review by journal staff. At this stage, your manuscript will be evaluated by a Science Editor with respect to its presentation of scientific content, compliance with journal policies, and presentation for a broad readership.

---

**Final acceptance from Science Editor March 31, 2022**

---
